# Graph Neural Network-Based Method of Spatiotemporal Land Cover Mapping Using Satellite Imagery

**DOI:** 10.3390/s23146648

**Published:** 2023-07-24

**Authors:** Domen Kavran, Domen Mongus, Borut Žalik, Niko Lukač

**Affiliations:** Faculty of Electrical Engineering and Computer Science, University of Maribor, Koroška Cesta 46, 2000 Maribor, Slovenia; domen.mongus@um.si (D.M.); borut.zalik@um.si (B.Ž.); niko.lukac@um.si (N.L.)

**Keywords:** multispectral, Sentinel-2, superpixel, node, EfficientNetV2, GraphSAGE

## Abstract

Multispectral satellite imagery offers a new perspective for spatial modelling, change detection and land cover classification. The increased demand for accurate classification of geographically diverse regions led to advances in object-based methods. A novel spatiotemporal method is presented for object-based land cover classification of satellite imagery using a Graph Neural Network. This paper introduces innovative representation of sequential satellite images as a directed graph by connecting segmented land region through time. The method’s novel modular node classification pipeline utilises the Convolutional Neural Network as a multispectral image feature extraction network, and the Graph Neural Network as a node classification model. To evaluate the performance of the proposed method, we utilised EfficientNetV2-S for feature extraction and the GraphSAGE algorithm with Long Short-Term Memory aggregation for node classification. This innovative application on Sentinel-2 L2A imagery produced complete 4-year intermonthly land cover classification maps for two regions: Graz in Austria, and the region of Portorož, Izola and Koper in Slovenia. The regions were classified with Corine Land Cover classes. In the level 2 classification of the Graz region, the method outperformed the state-of-the-art UNet model, achieving an average F1-score of 0.841 and an accuracy of 0.831, as opposed to UNet’s 0.824 and 0.818, respectively. Similarly, the method demonstrated superior performance over UNet in both regions under the level 1 classification, which contains fewer classes. Individual classes have been classified with accuracies up to 99.17%.

## 1. Introduction

Land cover mapping represents spatial information about the different types of the Earth’s surface coverage. Historically, one of the most influential causes of land cover changes is urbanisation [1,2]. Temporal observation of land cover changes is necessary for advanced crop [3] and disaster management (e.g., forest fires [4] and landslides [5]). Different land cover types have a heterogeneous regional impact on climate change [6]. This increases the demand for automatic land cover classification of geographically diverse regions further.

Breakthroughs in satellite control systems [7] and the development of techniques for well-organised formation flight above the Earth [8] have expanded the potential applications of satellite technology significantly. Satellite imagery, as a form of Big Data obtained with multispectral scanners, offers new perspectives in using remote sensing for land cover mapping and change detection [9,10,11]. Non-commercial satellites (e.g., Landsat, Sentinel) are a good alternative to commercial satellites for obtaining high spatial resolution imagery [12,13,14].

Several satellite imagery land cover datasets have been created. The Reunion Island dataset [15] consists of a time series of 21 Sentinel-2 images acquired in 2017. A respective pixel-wise ground truth map was built from various sources and completed by field experts [16]. Given the increasing desertification, rapid deforestation, abandonment of poor farmland, wetland drying, and continuous coastal urban development, there is an urgent need for comprehensive land cover inventories [17]. The Corine Land Cover (CLC) dataset [18], which provides pixel-wise ground truth maps of Europe and consists of 44 Land Use/Land Cover (LULC) classes, serves as an effective tool to manage these widespread environmental changes resulting from human activities [17]. The classification within the CLC is organised into three class levels: level 1 has 5 classes, level 2 contains 15 classes, and the most detailed, level 3, has 44 classes. The CLC inventory was initiated in 1985, and had the first release for the reference year of 1990 [19]. The inventory has been updated several times since, specifically in 2000 [20], 2006 [21], 2012 [22] and 2018 [23]. Throughout these years there have been significant improvements in the spatial resolution of the CLC dataset, from 50 m in the CLC1990 to 10 m in CLC2018, as well as the quality of satellite data used, transitioning from Landsat-5 images in 1990 to the use of Sentinel-2 and Landsat-8 images in 2018. The Land Use and Coverage Area frame Survey (LUCAS) [24], managed by Eurostat, has conducted in-depth surveys across Europe every three years since 2006, accumulating over 1 million data points from over 650,000 surveyed locations. These surveys provide rich data on the LULC, and agro-environmental information for supporting spatial, territorial and agricultural analyses. To address the dataset’s limitations and to make it more accessible, an open-source system was developed to integrate the LUCAS dataset into workflows [25]. A recently released dataset named Dynamic World NRT offers a global 10 m resolution LULC map with 9 classes [26].

Satellite imagery has been fused with derived data from other types of remote sensing, such as Light Detection and Ranging (LiDAR). The obtained data can be used further for creating digital 3-D models (e.g., terrain and cities). A city’s 3-D model and multispectral satellite data were used for spatial modelling of the land surface temperature [27]. Different combinations of imagery and Digital Elevation Models (DEMs) were used for landform classification using a deep learning-based approach [28].

New data sources and computational power have improved LULC mapping significantly using data from satellite imagery, land surveys and online portals. Different modelling techniques are used to predict LULC changes, including statistical models, cellular automata models (simulations that apply transition rules to study spatial dynamics/interactions), economic models, agent-based models (simulations of interactions of autonomous entities, e.g., landowners and policymakers), hybrid models that combine the strengths of individual models and time series modelling [29]. Machine Learning (ML) based methods are emerging as a powerful tool for LULC mapping by performing a pixel-based, a sub-pixel based or an object-based classification of satellite imagery [12]. The object-based classification is preferred, due to the high spatial resolution of satellite images. Several studies reported that the object-based approach produced better classification results compared to the pixel-based methods [30,31,32]. A specialised schema for enhancing the land cover identification [33] iteratively performs the assessment of the classification errors and lowers the error rate by creating new subclasses. Neural Networks (NNs) are the driving force in achieving state-of-the-art performance for land cover mapping, with Convolutional Neural Networks (CNNs) being the most frequently used architectures [34,35,36,37,38,39]. Recurrent Neural Networks (RNNs), especially Long Short-Term Memory (LSTM) networks, have shown a strong potential in utilising temporal information. This includes land cover classification [40,41], crop classification [42], predicting flood disasters [43] and performing natural disaster analysis [44]. Graph theory has achieved significant research advances [45,46,47] that have found applications across various industries and other research fields. Lately, researchers have started to utilise Graph Neural Networks (GNNs) for object-based land cover classification. GNN has been used in a method for 3-D building reconstruction, which used street view images and block meshes as inputs [48]. Researchers have proven the potential of Graph Attention Networks (GATs) [16] and fusion of aggregators in GNNs [49] to perform state-of-the-art land cover mapping as a node classification task.

In this paper, we present a novel spatiotemporal method for object-based LULC classification of satellite imagery using a GNN. The method starts with a superpixel segmentation, followed by segments being connected through time. Each individual segment is represented as a node in the graph. A target node (i.e., a selected segment) is classified by passing the subgraph with the target node and its neighbourhood through the classification pipeline. In the first step, the pipeline performs the image feature extraction with CNN. Importantly, the selection of the CNN architecture is not constrained to any specific requirements. This provides freedom to use any of the various state-of-the-art architectures, such as Vision Transformer (ViT) [50], EfficientNetV2 [51], EfficientNet [52], ResNet [53], ResNeXt [54], InceptionV3 [55], or DenseNet [56]. Following the extraction of features, the pipeline proceeds to the classification step, utilising a GNN to classify the target node. The choice of a GNN is as versatile as the CNN selection, offering options such as the Graph Attention Network (GAT) [57] or the SAmple and aggreGatE (GraphSAGE) framework [58]. This highlights the flexibility and modularity of the proposed method.

The main contributions of this paper, listed below, introduce novelties that have not been considered by the prior existing state-of-the-art ([16]):

- A new representation of the sequential satellite images as a directed graph by connecting segmented land region through time, based on sequential spatial segment overlaps.
- A new land cover mapping method as node classification in the derived directed graph using the GNN. The proposed method allows selection of a target node’s neighbourhood, which contains historical temporal context information of connected land region segments. The size of the neighbourhood determines the volume of input spatial and temporal information that the selected GNN uses for node classification.
- A modular target node classification pipeline, which offers flexible selection of a CNN for image feature extraction and a GNN for node classification.
- The first application of using EfficientNetV2 as a feature extractor for GraphSAGE classification models to perform intermonthly land cover classification.
- Complete intermonthly land cover classification maps for the given regions by using Sentinel-2 imagery, as shown in the Section 5.

The rest of this paper is organised as follows. Section 2 reviews related works. Section 3 presents the proposed land cover classification method. Dataset creation to evaluate the proposed method to the state-of-the-art is described in Section 4. The classification results and discussion are given in Section 5. Section 6 concludes this paper.

## 2. Related Work

In this section, we review related work in two categories: object-based land cover classification of satellite imagery and GNNs for land cover classification.

### 2.1. Object-Based Land Cover Classification of Satellite Imagery

A classification in hierarchical geospatial databases with a two-step deep learning framework has enabled simultaneous prediction of land use in all hierarchical levels [34]. This method achieved overall accuracies in the order of 90% for two datasets [34]. The main dataset contained satellite images of the settlements of Hameln and Schleswig in Germany with 8 land cover classes. State-of-the-art comparison was performed using the dataset from the ISPRS 2D semantic labelling challenge with satellite images of the settlements of Vaihingen and city of Potsdam in Germany with 6 land cover classes [59].

A multi-level context-guided method with the object-based CNN offers learning of high-level features from spectral patterns, geometric characteristics and object-level contextual information, based on a segmented context patch [32]. This method achieved remarkable accuracy (>80%) compared to traditional methods [32].

A semi-automated object-based land cover classification to observe changes in the Kurdistan region of Iraq using Landsat imagery was proposed [60]. After segmentation of the region, the spatial and spectral information were combined and followed by the object feature extraction. The classification was performed with a nearest neighbour classifier, achieving accuracies in the range of 88% to 89%.

A multi-view object-based classification with a CNN has proved to achieve a higher overall accuracy of 82% compared to application of a CNN on an orthoimage, which achieved an accuracy of 65% [61]. The results highlight that the window-based version of the proposed method can achieve better accuracy than the full object version.

Images from a multispectral camera, LISAT, onboard the third-generation satellite by LAPAN, named LAPAN-A3, were used in a study to perform object-based land cover classification of the Rote Island, using a tree method algorithm [62]. The results achieved overall classification accuracy of 86% for different classes, including open land, settlements, water bodies and diverse vegetation.

The study examined the potential of high-resolution PlanetScope data, made accessible on Google Earth Engine, to enhance land cover classification of Central Brazil, utilising both pixel-based and object-based methods [63]. The results revealed that the exclusive use of PlanetScope data yielded a 67% overall accuracy for pixel-based techniques and 82% for object-based methods. The fusion of PlanetScope data with Sentinel imagery data raised the overall accuracy to 82% for pixel-based and an impressive 91% for object-based methods.

A proposed method for paddy field mapping in Iran used rice phenology, yearly land surface temperature data and multi-temporal Landsat-8 imagery [64]. Enriched by a digital elevation model and spectral indices, the object-based classification outperformed the pixel-based approach with overall accuracy of 94% vs. 92%.

### 2.2. GNNs for Land Cover Classification

A Graph Convolutional Network (GCN) is a variant of CNNs which operates on graphs [65]. The layers in the model learn to encode both the local graph structure and the node features efficiently by performing approximation of the spectral convolution. Temporal GCN (T-GCN) is a combination of GCN and a Gated Recurrent Unit (GRU) to capture spatial and temporal dependencies simultaneously. T-GCN was designed originally for predicting traffic [66].

The Graph Attention Network (GAT) leverages masked self-attentional layers to help in extracting information from features of nodes [57]. The Symmetric Relation based Heterogeneous Graph Attention Network, denoted as SR-HGAT, is an extension of the original GAT network [67]. SR-HGAT takes the high-order relations of heterogeneous graphs into account [67]. A Robust Representation Learning method for multilabel images driven by GATs (RRL-GAT) reduces noisy and false connections between the connected image objects [68].

GraphSAGE (SAmple and aggreGatE) is a framework for generating inductive embeddings by sampling a neighbourhood of a node and then using a learnable aggregator function to aggregate features [58]. The data-efficient algorithm PinSAGE combines highly efficient random walks (improved upon GraphSAGE) and the graph convolutions for processing of graphs with billions of nodes [69].

A U-shaped object graph neural network (U-OGNN) for accurate land cover mapping using high-resolution remote sensing images was proposed [70]. The U-OGNN is composed of a self-adaptive graph construction that employs similarity measures to generate a contextual-aware graph structure. It also features a graph encoder and decoder, which fuse information across various scales, capturing hierarchical features of adjacent objects in images. The method demonstrated the overall accuracy of 88%.

A method for land cover classification, named AF2GNN, was proposed, using hyperspectral images as input to a GNN with adaptive filters and aggregator fusion [49]. Degree-scalers are defined to combine the multiple filters and present the graph structure. This method addresses the common problems of land cover discrimination, noise impact and spatial feature learning. The method achieved overall accuracies above 94% for various datasets.

An Attentive Spatial Temporal Graph CNN exploits the spatial and temporal information in satellite image time series in the context of land cover mapping [16]. After performing the segmentation with the SLIC algorithm [71], the graph is formed by connecting the adjacent segments. Each georeferenced segment or node contains a time series of segments. Target and neighbourhood segments are passed to separate inputs of the classification NN, which utilises the attention mechanism. The NN outputs the classification of the target segment. To the best of our knowledge, this is the first and only spatiotemporal application of GNNs for remote sensing data.

The input graphs into the Attentive Spatial Temporal Graph CNN represent connected segments based on an adjacency in the space without using any kind of temporal information [16]. This method obtained the graphs by segmenting a hand-picked time series image that experts considered suitable. The proposed method in this paper takes shifting and growing of segments into account additionally. The thresholded temporal connections between segments are formed, based on sequential spatial segment overlaps in segmented time series images. The GNN in the proposed method can be replaced by any chosen GNN algorithm, making the proposed method modular, as opposed to the Attentive Spatial Temporal Graph CNN [16], which uses the attention mechanism from GATs exclusively.

## 3. Methodology

**Figure 1:**
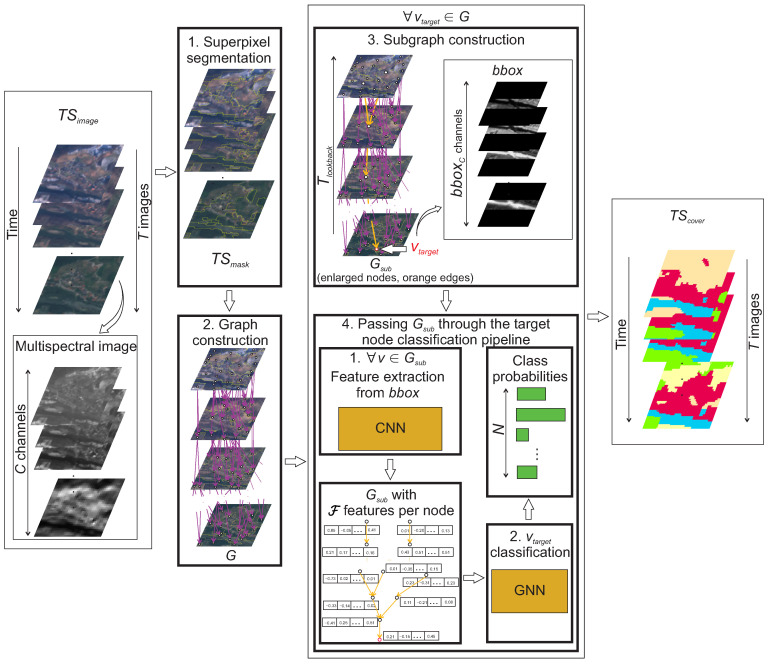
The proposed method’s workflow with four main steps.

This paper proposes a novel spatiotemporal method for the LULC classification of satellite imagery using a GNN. The workflow of the proposed method is shown in Figure 1. The proposed method starts by performing superpixel segmentation (step 1 in Figure 1) on each multispectral image in the time series of satellite images $TS_{image}$ of length *T*. This results in a time series of segmentation masks $TS_{mask}$. The next step is the graph construction (step 2 in Figure 1), based on temporal segment overlaps, where each segment in each satellite image is represented as a node *v*. An edge between two nodes is made if two segments in two sequential satellite images overlap sufficiently. The constructed graph *G* contains the same number of nodes as the number of all superpixels in $TS_{mask}$. Each *v* contains the segment representation as a resized multispectral image bounding box, bbox. Next, a subgraph $G_{sub}$ is created for each target node $v_{target}$ based on the input edges towards it (step 3 in Figure 1). The $v_{target}$ represents the selected segment $s_{selected}$ to classify. The $G_{sub}$ is then passed into the target node classification pipeline (step 4 in Figure 1). It performs feature extraction from bbox with any chosen CNN (step 4.1 in Figure 1). This is followed by $v_{target}$ classification with a selected GNN (step 4.2 in Figure 1), which outputs the class probabilities. The land cover class of $s_{selected}$ is determined based on the highest probability. The final result is the time series of the land cover mappings $TS_{cover}$.

The following subsections provide detailed descriptions of all four main steps of the proposed method.

### 3.1. Superpixel Segmentation

Superpixels are connected groups of similar pixels that form a visually meaningful entity while reducing the number of primitives for subsequent processing. This makes many algorithms computationally feasible on high dimensional images. Pixels are grouped by colour, proximity and other low-level properties [72].

In the proposed method, a multispectral image with *C* layers is segmented to create meaningful land segments, which surround objects, e.g., buildings, field crops, forests, rivers and roads. The superpixels are calculated with Felzenszwalb’s efficient graph based image segmentation [73]. The algorithm has three parameters [73]:

- σ—the Standard Deviation of the Gaussian kernel to smooth the image in the preprocessing stage;
- *k*—a scale of observations for the threshold function, which controls the degree of required difference between two adjacent superpixels (a larger *k* causes a preference for larger superpixels);
- min_size—the minimum number of pixels inside the superpixel to control the merging of neighbouring superpixels in the postprocessing stage.

The result of segmenting a single satellite image, captured at time *t*, is a segmentation mask $mask_{t}$, which is the *t*-th mask in $TS_{mask}$.

**Figure 2:**
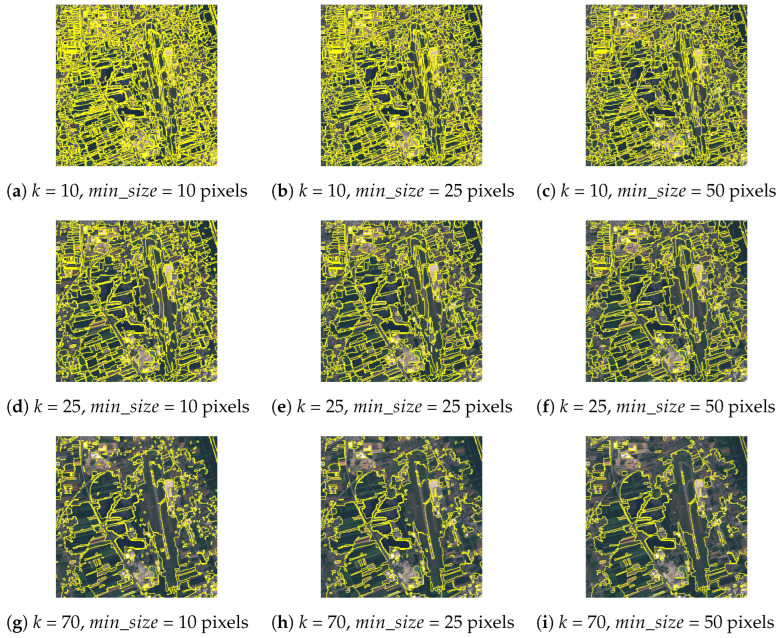
Examples of applying Felzenszwalb’s image segmentation algorithm (σ = 0.5) on a Sentinel-2 13-layer image with 10 m spatial resolution of an example region of Graz, Austria in July of 2017. A True colour (RGB) composite is shown of the multispectral image.

The application of Felzenszwalb’s image segmentation algorithm is demonstrated in Figure 2. The segmentation masks with *k* = 70 reveal that large superpixels (i.e., segments) enclose too many meaningful land objects. The results from a small *k* value contain too many small segments, which leads directly to higher computational cost in the later steps of the method. The value of min_size should be adjusted accordingly, to avoid the presence of the remaining small segments in heterogeneous areas of the image.

### 3.2. Graph Construction

**Figure 3:**
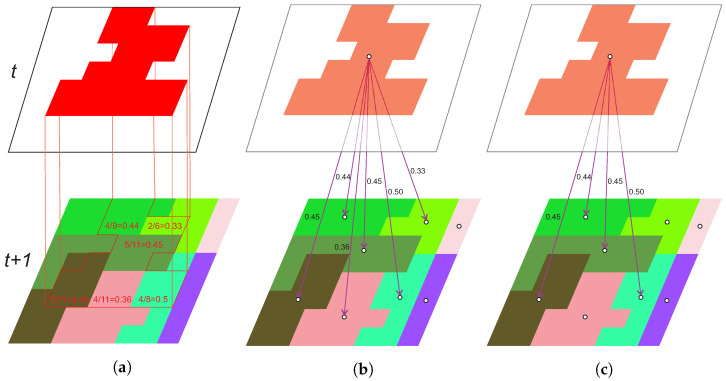
An example of overlap portion calculations between segments, shown in (**a**), and creation of *G* with τ = 0.3 in (**b**) and τ = 0.4 in (**c**). (**a**) Overlap portion calculations between the red segment in time *t* and colored segments in time t+1, (**b**) τ = 0.3, (**c**) τ = 0.4.

The $TS_{mask}$ is the fundamental for constructing a weighted directed graph *G*, connecting segments through time. Each *i*-th segment *s* from the $mask_{t}$, labelled as $s_{i,t}$, is added as a node $v_{i,t}$ in *G*. The created *G* contains the same amount of nodes as there are segments in $TS_{mask}$. An individual $v_{i,t}$ forms a directed edge to $v_{j,t+1}$, if $s_{i,t}$ overlaps at least τ (the value range is the (0,1.0]) portion of the $s_{j,t+1}$. The overlap portion serves as the weight $w_{i,j}$ of the formed edge. An example of graph construction is shown in Figure 3.

### 3.3. Segment Representation and Subgraph Construction

An individual v∈G holds the corresponding segment’s multispectral image bounding box, bbox. It can be extended in width and height to capture additional spatial contextual information around the segment. Due to segments being of different shapes and, consequently, the bounding boxes being of various sizes, the resizing of each bbox must be performed to the width $bbox_{w}$ and height $bbox_{h}$. The resizing keeps the original aspect ratio of the multispectral image inside the bbox by zero padding. This resizing is needed to ensure that all data inside the nodes are of equal size. It is worth noting that bbox contains a $bbox_{C}$ number of layers.

The target node $v_{target}$ (i.e., the selected segment $s_{selected}$) and it’s neighbourhood of nodes in *G* form a subgraph $G_{sub}$. The parameter $T_{lookback}$ controls the historical temporal context information in $G_{sub}$ by determining the maximum distance of the nodes, based on the input edges towards the $v_{target}$. By setting the value of $T_{lookback}$ = 0, only the $v_{target}$ at time *t* will be present in the constructed $G_{sub}$. By increasing the value of $T_{lookback}$ to, e.g., 2, the $G_{sub}$ will feature the $v_{target}$ at time *t*, all nodes at time t−1 with directed edges towards the $v_{target}$, and all nodes at time t−2 with edges directed towards all previously included nodes in time t−1.

**Figure 4:**
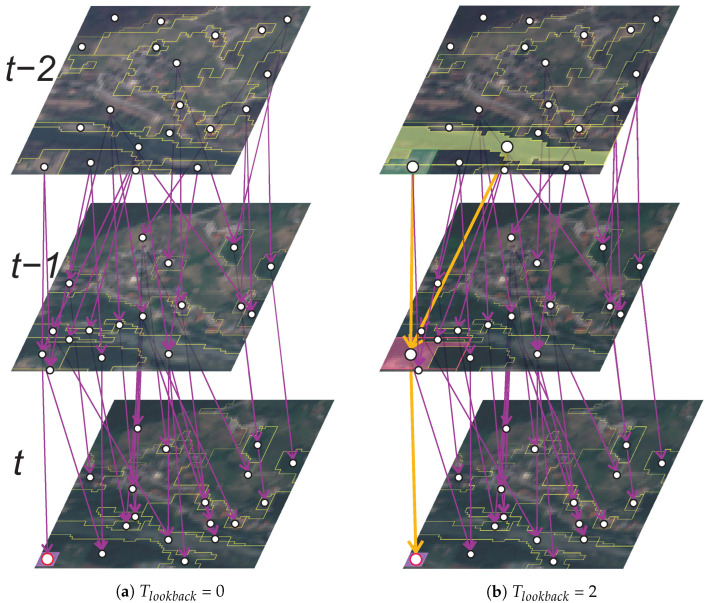
Examples of $G_{sub}$ construction for a $v_{target}$ (red) in time *t* at $T_{lookback}$ = [0, 2]. The *G* was constructed for a $TS_{mask}$ with 3 images of a small example subregion using τ = 0.2. The orange edges connect all the included (enlarged) nodes in the $G_{sub}$. Each included *v* has a bbox drawn around the coloured *s* it represents. $G_{sub}$ in (**a**) includes only the $v_{target}$, and the $G_{sub}$ in (**b**) contains $v_{target}$ and 3 nodes with 3 edges between them.

The largest subgraphs can be constructed for the leaf nodes of *G*, which represent segments obtained for the final image in $TS_{satellite\text{\_}image}$ at time *t* = *T*. Even though $T_{lookback}$ can be of any number greater than or equal to 0, each $G_{sub}$ can contain nodes and edges only up to the first image at *t* = 0. An important fact is that subgraphs of all target nodes in *t* = 0 actually contain only the $v_{target}$ itself, because the nodes at time *t* = 0 do not contain any input edges. The $G_{sub}$ construction is demonstrated in Figure 4. All the visualised subgraphs have bounding boxes drawn around the included nodes.

### 3.4. Node Classification Pipeline

**Figure 5:**
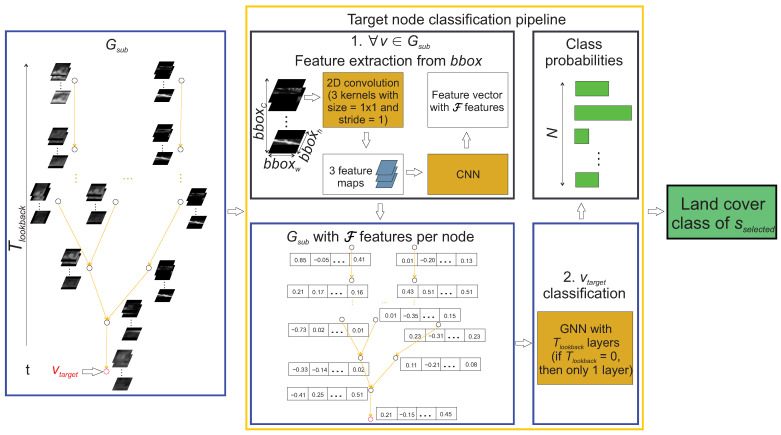
Target node classification pipeline, which outputs the land cover class of the $s_{selected}$ by classifying the $v_{target}$ based on the input $G_{sub}$.

The $G_{sub}$ serves as the input to the target node classification pipeline, which outputs the classification probabilities of the $s_{selected}$, represented by the $v_{target}$. The node classification pipeline is visualised in Figure 5. The first step is the feature extraction, which is performed $\forall v\;\in \;G_{sub}$ (step 1 in Figure 5). Due to the bbox inside each *v* containing a $bbox_{C}$ number of layers and the majority of state-of-the-art CNNs requiring a 3 channel input, the bbox is first passed through the 2D convolution with 3 kernels of size 1 × 1 and a stride of 1. This converts the depth of the input from $bbox_{C}$ to 3. The 3 feature maps are then passed to the CNN. It extracts F features in the form of a feature vector, which is then stored inside the *v*. The value of F depends on the output size of the last feature extraction layer of the CNN (the softmax classification layer at the end of NN is removed). For majority of established CNNs, the number of output features from last feature extraction layer cannot be adjusted without modifying previous layers of the NN. After feature extraction, the $G_{sub}$ is passed as the input to the GNN (step 2 in Figure 5). It contains the same number of layers as the value of $T_{lookback}$ (the only exception being the case of $T_{lookback}$ = 0 with GNN containing a single layer). The GNN outputs *N* class probabilities of $v_{target}$. The class of $s_{selected}$ is determined as the class with the highest probability. After the classification of $\forall v_{target}\;\in \;G$, the time series is created of land cover mappings $TS_{cover}$.

## 4. Dataset Preparation

Datasets with LULC classification of satellite imagery on a daily/weekly/monthly basis for a multi-year period of time are not available. This section describes creation of an intermonthly dataset to evaluate and compare the performance of the proposed method to the state-of-the-art. The first Subsection provides details about acquisition of Sentinel-2 images of the selected regions. The second Subsection describes the process of creating land cover ground truth.

### 4.1. Intermonthly Satellite Imagery Acquisition

To create the land cover dataset, the first step was to acquire atmospherically corrected Sentinel-2 L2A images, with the earliest globally available being from 2017. From 1992 to 2015 the conversion of cropland to artificial surfaces was notably high in Austria and Slovenia, with rates above 4% and 3% respectively, exceeding the average rate of above 2% observed across the European Union (EU28). In the same period, Austria and Slovenia also underwent a conversion of (semi-)natural vegetated areas to cropland at rates above 2% and nearly 6% respectively, with the EU28 average just under 3% (https://www.oecd.org/environment/indicators-modelling-outlooks/monitoring-land-cover-change.htm (accessed on 12 July 2023)). Given these trends, a region of Graz in Austria, and the region of the cities—Portorož, Izola and Koper—in Slovenia were selected, due to their spatial heterogeneity.

Images were obtained for the two selected regions: Graz, Austria, encapsulated within the WGS84 bounding box [15.390816°, 46.942176°, 15.515785°, 47.015961°], and the region of Portorož, Izola and Koper in Slovenia, defined by the bounding box [13.590260°, 45.506948°, 13.744411°, 45.554449°]. These values denote pairs of minimum and maximum longitudes and latitudes, respectively. The created $TS_{image}$ for Graz contained 40 intermonthly Sentinel-2 multispectral images with minimal cloud coverage, spanning from January 2017 to July 2021. Similarly, the $TS_{image}$ for the region of Portorož, Izola and Koper contained 41 intermonthly Sentinel-2 multispectral images, also characterised by minimal cloud coverage, captured from January 2017 through June 2021. Months with too high cloud coverage were skipped.

**Figure 6:**
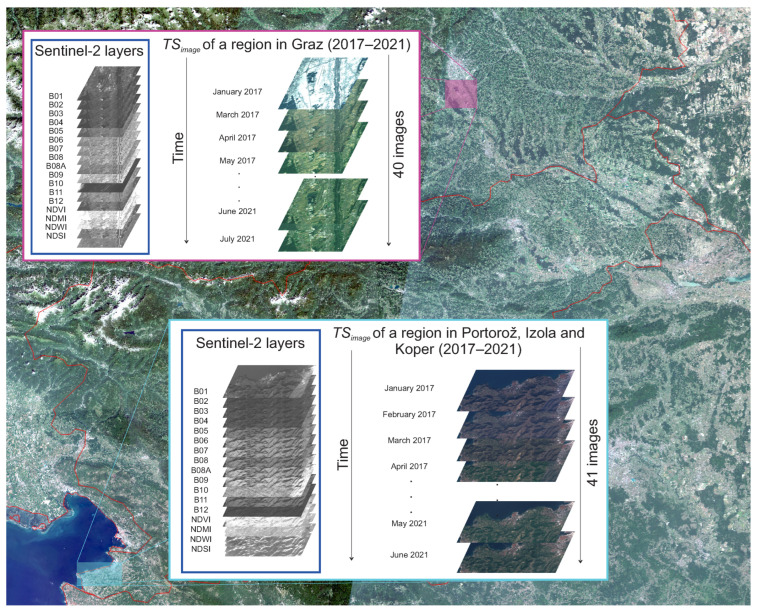
Intermonthly $TS_{image}$ for the region of Graz and the region of Portorož, Izola and Koper. The individual multispectral image contains *C* = 17 layers. The images in $TS_{image}$ are visualised with a True colour (RGB) composite.

Each image stores data as unsigned 16-bit integers. The images for the Graz region have the size of 946 × 825 pixels, corresponding to an area of 78 km${}^{2}$ at a 10 m spatial resolution, while those for the region of Portorož, Izola and Koper are 1212 × 508 pixels in size, representing an area of 61 km${}^{2}$ at the same spatial resolution. All images contain 13 basic spectral layers (B01–B12) and 4 calculated layers: Normalised Difference Vegetation Index (NDVI), Normalised Difference Moisture Index (NDMI), Normalised Difference Water Index (NDWI) and Normalised-Difference Snow Index (NDSI). The spatial resolution of layers ranged from 10 m to 60 m. The described $TS_{image}$ for both regions are visualised in Figure 6.

### 4.2. Land Cover Ground Truth Creation

The next step of dataset creation was the superpixel segmentation. Segmentation masks serve as the basis for creation of the time series of ground truth land cover mappings $TS_{gt\text{\_}cover}$. An individual image was segmented, based on the normalised values of 13 basic spectral layers. The Felzenszwalb’s segmentation algorithm used the parameters σ = 0.5, *k* = 12 and min_size = 15 pixels. These values were selected empirically, to ensure a balanced tradeoff between segments being small enough to capture complex land objects and the total number of segments not exceeding the storage limitations of hardware that we used for deep learning.

**Figure 7:**
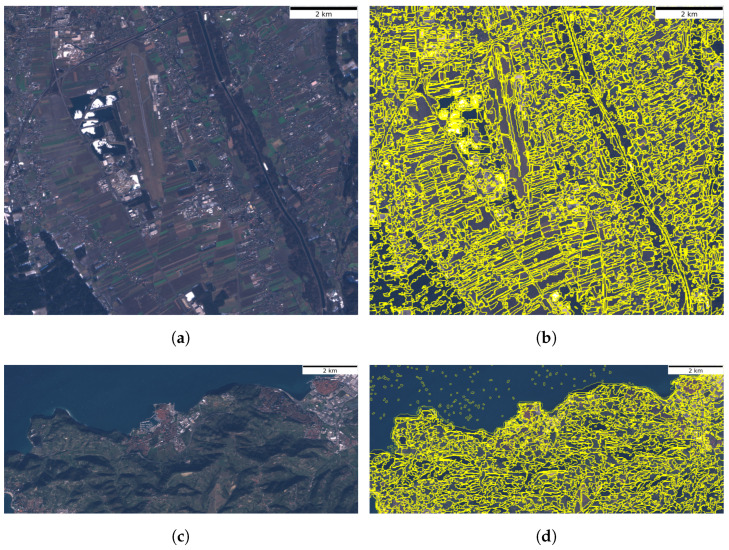
Examples of the segmented regions. Images (**a**,**c**) show the True colour (RGB) composites, while (**b**,**d**) show their respective segmentation masks. (**a**) The region of Graz in January 2019, (**b**) The mask for the region of Graz in January 2019, (**c**) The region of Portorož, Izola and Koper in November 2018, (**d**) The mask for the region of Portorož, Izola and Koper in November 2018.

The created $TS_{mask}$ for the region of Graz contains 224,665 segments (avg. 5616 segments/mask). Similarly, the $TS_{mask}$ for the region of Portorož, Izola and Koper has 122,685 segments (avg. 2992 segments/mask). Examples of the segmentation results are visible in Figure 7.

The last step of dataset creation was the ground truth land cover labelling. For each dataset region, an individual *s* of $\forall mask\in TS_{mask}$ was labelled with a single class among 15 LULC classes in CLC2018 level 2 [18]. To speed up the labelling process, we utilised a pretrained UNet model by Esri (https://www.arcgis.com/home/item.html?id=afd124844ba84da69c2c533d4af10a58 (accessed on 12 July 2023)), which achieved an overall pixel-based accuracy of 84.0% for CLC level 2 classification. A multispectral image with 13 basic spectral layers served as the input to the pretrained model. Before passing the image to the UNet model, each layer had to be normalised and standardised, based on provided pretrained model statistics. The last step of preprocessing was scaling of the input image to 1280 × 1280 pixels (nonsquare images were zero padded). The input image was then passed into the UNet model, which output a tensor of size 1280 × 1280 × 15 (15 CLC level 2 classes). The output tensor was reshaped into a size of 1280 × 1280 × 1 by performing the argmax function across the 3rd axis. The classification tensor was then reshaped to match the size of the original image by downscaling and removal of the padding. The classification tensor and respective mask were then used to create a ground truth image. This was performed automatically by selecting each individual *s* from mask and assigning it the majority class of associated pixels from the classification tensor. The automatically created ground truth image was finalised with manual label corrections of segments. This was necessary due to rare misclassifications from the UNet model. By performing the described process on all images in individual $TS_{image}$, the time series was created of the ground truth land cover mappings $TS_{gt\text{\_}cover}$.

**Figure 8:**
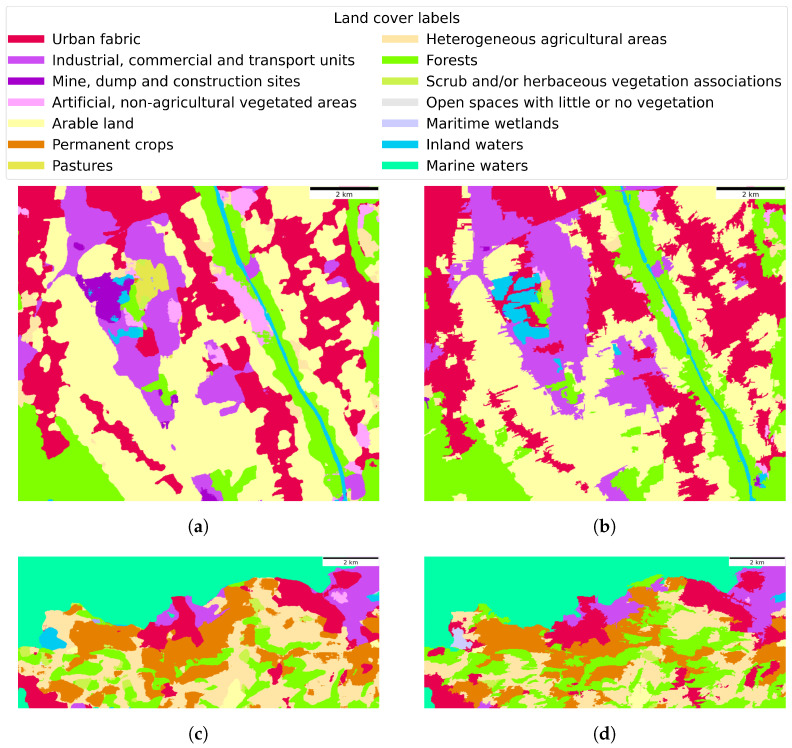
Examples of CLC level 2 classification outputs, obtained with the UNet model by Esri, are shown in (**a**,**c**). The manually corrected ground truth, derived from respective UNet model outputs, are shown in (**b**,**d**). (**a**) Classification output of Esri’s UNet model for the region of Graz in January 2019, (**b**) Manually corrected ground truth for the region of Graz in January 2019, (**c**) Classification output of Esri’s UNet model for the region of Portorož, Izola and Koper in November 2018, (**d**) Manually corrected ground truth for the region of Portorož, Izola and Koper in November 2018.

The $TS_{gt\text{\_}cover}$ for the region of Graz contains 12 of the 15 CLC level 2 classes, excluding Inland Wetlands, Maritime wetlands, and Marine waters; meanwhile, the $TS_{gt\text{\_}cover}$ for the region of Portorož, Izola and Koper includes 13 out of the 15 classes, omitting Mine, dump and construction sites, and Inland wetlands from the selected region. Examples of classification output, obtained with the UNet model, and manually corrected time series of the ground truth $TS_{gt\text{\_}cover}$, are visualised in Figure 8.

**Figure 9:**
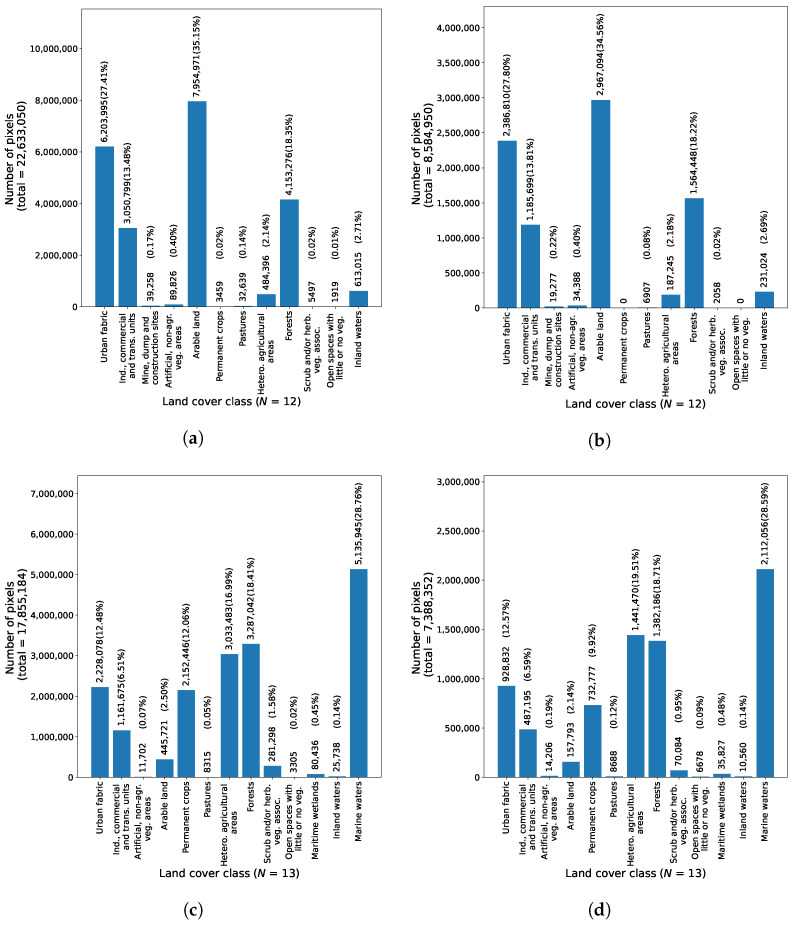
Number of pixels per land cover class for each region in the dataset. Images (**a**,**c**) show the class distribution in $TS_{train\text{\_}gt\text{\_}cover}$, while (**b**,**d**) show the class distribution in $TS_{test\text{\_}gt\text{\_}cover}$. (**a**) Distribution of ground truth land cover labels of pixels in $TS_{train\text{\_}gt\text{\_}cover}$ for the region of Graz, (**b**) Distribution of ground truth land cover labels of pixels in $TS_{test\text{\_}gt\text{\_}cover}$ for the region of Graz, (**c**) Distribution of ground truth land cover labels of pixels in $TS_{train\text{\_}gt\text{\_}cover}$ for the region of Portorož, Izola and Koper, (**d**) Distribution of ground truth land cover labels of pixels in $TS_{test\text{\_}gt\text{\_}cover}$ for the region of Portorož, Izola and Koper.

For each region, the $TS_{image}$ was split into $TS_{train\text{\_}image}$ and $TS_{test\text{\_}image}$. Specifically, the Graz region’s $TS_{image}$ was split into a $TS_{train\text{\_}image}$ that containes 29 consecutive images (accounting for 72.5% of all images) from January 2017 to April 2020, and a $TS_{test\text{\_}image}$ with 11 consecutive images (making up 27.5% of all images) from May 2020 to July 2021. Similarly, the $TS_{image}$ for the region of Portorož, Izola and Koper was split into a $TS_{train\text{\_}image}$, containing 29 consecutive images (70.7% of all images) from January 2017 to October 2019, and a $TS_{test\text{\_}image}$, containing 12 consecutive images (29.3% of all images) from January 2020 to June 2021. The split ratio was decided upon by the fact that each $TS_{test\text{\_}image}$ should contain images across more than a year. The $TS_{gt\text{\_}cover}$ and $TS_{mask}$ of each region were split in the same manner into $TS_{train\text{\_}gt\text{\_}cover}$ and $TS_{test\text{\_}gt\text{\_}cover}$, as well as $TS_{train\text{\_}mask}$ and $TS_{test\text{\_}mask}$. The distribution of ground truth land cover labels of pixels in individual $TS_{train\text{\_}gt\text{\_}cover}$ and $TS_{test\text{\_}gt\text{\_}cover}$ are shown in Figure 9. Note that $TS_{test\text{\_}gt\text{\_}cover}$ for the region of Graz lacks pixels with land cover classification of Permanent crops and Open spaces with little or no vegetation.

## 5. Results and Discussion

The proposed method was evaluated on the created dataset, with its performance compared against that of the current state-of-the-art. The first Subsection describes the application of the proposed method on the created dataset. The second Subsection describes the analysis of GNN usage in the proposed method, while the classification performance and discussion are presented in the third Subsection.

### 5.1. Application of the Proposed Method and Experimental Parameters

From the dataset creation process, the $TS_{train\text{\_}mask}$ and $TS_{test\text{\_}mask}$ of both regions were carried over. For each individual region in the dataset, the $TS_{train\text{\_}image}$, $TS_{train\text{\_}mask}$ and $TS_{train\text{\_}gt\text{\_}cover}$ were used for constructing the training graph $G_{train}$, with $TS_{test\text{\_}image}$, $TS_{test\text{\_}mask}$ and $TS_{test\text{\_}gt\text{\_}cover}$ being used to construct the test graph $G_{test}$. The construction of both graphs for individual region was performed using the empirically set parameter τ = 0.2. This value ensured that the number of inbound edges per node was limited to 5.

**Figure 10:**
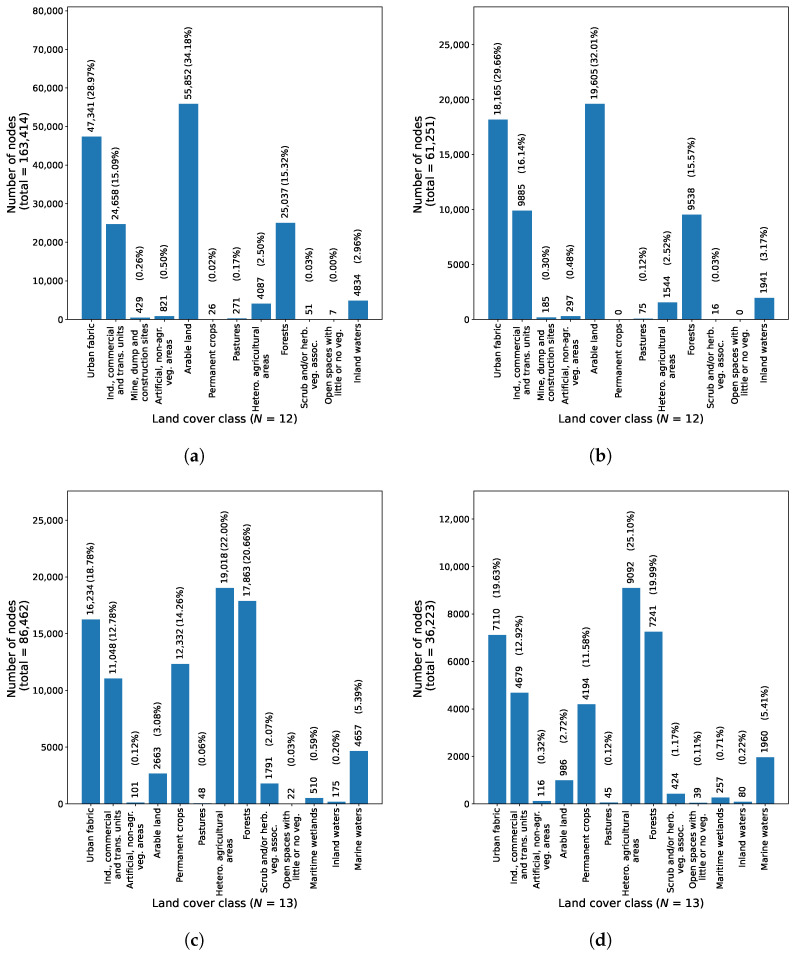
Number of nodes per land cover class for each region in the dataset. Images (**a**,**c**) shows the class distribution in $G_{train}$, while (**b**,**d**) show the class distribution in $G_{test}$. (**a**) Distribution of ground truth land cover labels of nodes in $G_{train}$ for the region of Graz, (**b**) Distribution of ground truth land cover labels of nodes in $G_{test}$ for the region of Graz, (**c**) Distribution of ground truth land cover labels of nodes in $G_{train}$ for the region of Portorož, Izola and Koper, (**d**) Distribution of ground truth land cover labels of nodes in $G_{test}$ for the region of Portorož, Izola and Koper.

For the region of Graz, the constructed $G_{train}$ contained 163,414 nodes, and the $G_{test}$ had 61,251 nodes. Similarly, for the region of Portorož, Izola and Koper, the $G_{train}$ had 86,462 nodes and the $G_{test}$ 36,223 nodes. The distribution of ground truth land cover classification of nodes in $G_{train}$ and $G_{test}$ for both dataset regions is shown in Figure 10.

For the classification of individual region within the created dataset, we used the following set of experimental parameters:

- Individual *v* contained the bbox, which was extended by 20 pixels in both width and height. The bbox was resized to the size of $bbox_{w}$ = 48 and $bbox_{h}$ = 48. It also contained $bbox_{C}$ = 7 layers, specifically, B04, B03, B02, NDVI, NDMI, NDWI and NDSI.
- The feature extraction part of the target node classification pipeline starts by passing the segment’s bbox through the trainable 2D convolution with 3 kernels. This outputs a tensor with 3 feature maps. These are then passed into the CNN - the state-of-the-art EfficientNetV2-S [51] was selected. It had the fully-connected output layer removed and all the layers trainable. Before passing the 3 feature maps into the EfficientNetV2-S, they were passed through the EfficientNetV2-S’s preprocessing transforms. The final output of the EfficientNetV2-S was F = 1280 extracted features.
- Each but the last layer in the GNN had a hidden dimension of 256, with a dropout of 0.5. The output dimension of the final layer in the GNN was set to match the number of classification classes *N*. Specifically, it was set to 12 for the Graz region and 13 for the region of Portorož, Izola and Koper. All the GNN layers were trainable.
- Before training, the EfficientNetV2-S model for feature extraction was initialised with ImageNet pretrained weights, because the preliminary experiments showed that this led to lower training loss. The classification pipeline forms a single model and it was, same as in [58], trained in a single training process using the Adam optimiser [74]. The initial learning rate was set to 0.001 and the model was trained for 10 epochs with a random shuffle of the training samples (i.e., subgraphs) inbetween. The batch size was 30. The loss function was categorical cross-entropy, which used class weights, due to unbalanced ground truth land cover labels of the nodes in $G_{train}$. The edge weights in $\forall G_{sub}$ were ignored during the message passing through the GNN, because initial empirical experiments had shown that that led to lower training loss.

Regarding the experimental protocol, for each region in the dataset the experiments were performed for multiple values of $T_{lookback}$. A total of 10 runs were performed per experiment.

Overall accuracy reflects correct classification for all classes combined, while the class-specific accuracy assesses the correctness for each individual class independently [75]. The equations for the *i*-th class accuracy and overall accuracy are defined with (1) [76]. The equations contain $TP_{i}$, $TN_{i}$, $FP_{i}$ and $FN_{i}$, to denote True Positives (the number of samples identified correctly as positive), True Negatives (the number of samples identified correctly as negative), False Positives (the number of samples identified incorrectly as positive) and False Negatives (the number of samples identified incorrectly as negative) for the *i*-th class, respectively. Recall (or sensitivity) quantifies how many actual positive instances were identified correctly [76]. Precision measures the proportion of true positive predictions among all the positive predictions [76]. The F1-score is the harmonic mean of precision and recall, providing a balance between precision and recall, making it especially useful for imbalanced datasets [76]. All three class-specific metrics are defined with (2) [75]. The metrics can be micro-averaged (all samples contribute equally), macro-averaged (all classes constribute equally) and weighted-averaged (the contribution of each class is weighted by the proportion of its instances in the total data, labelled as $w_{i}$). The equations for micro- and macro-averaged recall, precision and F1-score are defined with (3), (4) and (5), respectively [75]. The weighted-averaged variants of the metrics are defined with (6) and (7) [75]. The choice between micro-, macro-, and weighted-averaging depends on the balance of the dataset and the problem that is being solved. Micro-averaging is the preferred approach if every sample in the dataset holds equal importance. On the other hand, macro-averaging is used when the significance is distributed uniformly among all the classes. Finally, weighted-averaging should be used with imbalanced datasets where the relevance of classes is defined by their size [75]. Given the significant imbalance in the created dataset, as previously shown in Figure 10, the weighted F1-score alongside accuracy were used as the primary evaluation metrics.

(1) $$Accuracy_{i}=\frac{TP_{i}+TN_{i}}{TP_{i}+FP_{i}+FN_{i}+TN_{i}},\;\;Accuracy=\frac{\sum_{i}TP_{i}+TN_{i}}{\sum_{i}\left(TP_{i}+FP_{i}+FN_{i}+TN_{i}\right)}$$

(2) $$Recall_{i}=\frac{TP_{i}}{TP_{i}+FN_{i}},\;\;Precision_{i}=\frac{TP_{i}}{TP_{i}+FP_{i}},\;\;F1_{i}=\frac{2\cdot Precision_{i}\cdot Recall_{i}}{Precision_{i}+Recall_{i}}$$

(3) $$MicroRecall=\frac{\sum_{i}TP_{i}}{\sum_{i}\left(TP_{i}+FN_{i}\right)},\;\;MacroRecall=\frac{1}{N}\sum_{i}Recall_{i}$$

(4) $$MicroPrecision=\frac{\sum_{i}TP_{i}}{\sum_{i}\left(TP_{i}+FP_{i}\right)},\;\;MacroPrecision=\frac{1}{N}\sum_{i}Precision_{i}$$

(5) $$MicroF1=\frac{2\cdot MicroPrecision\cdot MicroRecall}{MicroPrecision+MicroRecall},\;\;MacroF1=\frac{1}{N}\sum_{i}F1_{i}$$

(6) $$WeightedRecall=\sum_{i}w_{i}\;Recall_{i},\;\;WeightedPrecision=\sum_{i}w_{i}\;Precision_{i}$$

(7) $$WeightedF1=\sum_{i}w_{i}\;F1_{i}$$

### 5.2. Analysis of GNN Usage in the Proposed Method

In order to choose the best performing GNN for the proposed method’s target node classification pipeline, the experiments were performed on a subset of the created dataset. More precisely, only nodes in $G_{train}$ from the first seven satellite images (from January 2017 to August 2017) were used to train classification models, to classify the $G_{test}$ of the region of Portorož, Izola and Koper. The experimental values for $T_{lookback}$ were 〈0,1,2,3〉. The average accuracy and weighted F1-score were calculated for the CLC classification at class level 2 considering 10 runs. Two GNNs were considered for this evaluation:

- The GraphSAGE algorithm [58], which applied LSTM aggregation in each of its layers. The output of the hidden layers was passed through the rectified linear unit (ReLU) activation function, as it was done in [58].
- GAT [57] with outputs of hidden layers being passed through the exponential linear unit (ELU) activation function.

In addition, we also examined the effect of eliminating the GNN from the target node classification pipeline. In this case, global average pooling was performed for each individual extracted feature across all nodes in the subgraph $G_{sub}$. More specifically, feature vectors from all nodes in each $G_{sub}$ were stacked in a tensor with 1280 columns. Each individual feature then underwent global average pooling, which resulted in a tensor of size 1 × 1280. Following this process, an output fully connected layer was utilised, receiving the tensor as input and yielding the output classification tensor with size equal to the number of classes *N*. Importantly, when $T_{lookback}$ = 0 and the GNN is not used, the whole classification process essentially becomes an image classification task using EfficientNetV2-S. The experiments of excluding a GNN from the target node classification pipeline were conducted, to determine the necessity of using a GNN in the proposed method to achieve high overall classification performance.

**Figure 11:**
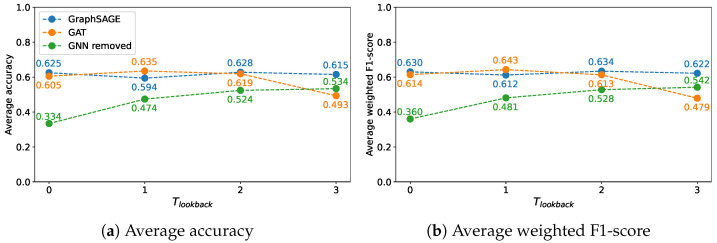
CLC level 2 classification results for a region of Portorož, Izola and Koper, depending on the selection of GNN and the value of $T_{lookback}$.

The results displayed in Figure 11 demonstrate how both the choice of GNN and the value of $T_{lookback}$ affect the performance of the proposed method for the CLC level 2 classification. Based on the obtained results, utilising GraphSAGE as the GNN in the target node classification pipeline yielded the highest performance for $T_{lookback}$ values of 0, 2, and 3. Specifically, these values resulted in average accuracies of 0.625, 0.628 and 0.615, and average weighted F1-scores of 0.630, 0.634 and 0.622, respectively. However, an exception was observed at a $T_{lookback}$ value of 1, where GraphSAGE did not exhibit the best performance, resulting in a decrease in average accuracy to 0.594 and weighted F1-score to 0.612. At that specific value of $T_{lookback}$, the GAT outperformed GraphSAGE, achieving an average accuracy of 0.635 and a weighted F1-score of 0.643. Notably, there was a more significant decline in performance for GAT compared to GraphSAGE, particularly at a $T_{lookback}$ value of 3. This observation suggests that GAT is more sensitive to higher values of $T_{lookback}$, resulting in a larger drop in classification performance.

Removal of the GNN entirely from the proposed method led to the worst performance across all tested $T_{lookback}$ values. However, as the value of $T_{lookback}$ increased, there was a noticeable improvement in the results. The average accuracy improved from 0.334 to 0.534, while the average weighted F1-score increased from 0.360 to 0.542. These results demonstrate the necessity of utilising a GNN to achieve high classification performance with the proposed method. GraphSAGE demonstrated its superiority as the best-performing GNN.

### 5.3. Classification Performance and Discussion

Based on the results from the previous subsection, the GraphSAGE algorithm, utilising LSTM aggregation, showcased the highest performance among all the evaluated GNNs. Hence, it was chosen as the GNN within the target node classification pipeline for the complete performance evaluation of the proposed method, using both regions in the dataset. Unlike with training in the prior subsection, all nodes in each $G_{train}$ were utilised to train the classification models. The experiments were performed for $T_{lookback}$ = 〈0,1,…,7〉 for each region in the dataset. The average and Standard Deviation of accuracy, precision, recall and F1-score were calculated for the CLC classification at both class levels 1 and 2 considering all runs. The level 1 classification results were derived by using the level 2 results and combining them according to the predefined class structure of level 1, as defined in the CLC dataset [18].

The training for both regions was undertaken on two distinct platforms. For the region of Graz, a dedicated Deep Learning server was used, equipped with two Intel Xeon E5-2683 v4 processors, 98GB of RAM, and four NVIDIA GeForce GTX TITAN X graphics cards. Here, the average epoch training time varied from 18 min for $T_{lookback}$ = 0 to 64 min for $T_{lookback}$ = 7. The training times for 10 runs at $T_{lookback}$ = 〈0,1,…,7〉 were 30, 40, 41, 44, 56, 70, 88 and 107 h, respectively. The total training time was 476 h.

In contrast, the region of Portorož, Izola and Koper was trained on a personal computer powered by an AMD Ryzen 9 7900X processor, 64GB of RAM and an NVIDIA GeForce RTX 4090 graphics card. The average epoch training time was shorter, from 2 min at $T_{lookback}$ = 0, extending to 12 min for $T_{lookback}$ = 7. The accumulated training times for 10 runs at $T_{lookback}$ = 〈0,1,…,7〉 were reduced considerably, specifically to 3, 4, 6, 8, 10, 13, 17 and 21 h, respectively. This sums up to a total of 82 h of training time.

The pixel-based CLC level 2 classification results of the $TS_{test\text{\_}image}$, for both dataset regions, are depicted using various metrics in Table 1 and Table 2. For the Graz region, the Esri’s UNet model achieved an accuracy of 0.818, weighted precision of 0.841, weighted recall of 0.818 and weighted F1-score of 0.824. The proposed GNN-based method achieved better performance across all metrics, but at various sizes of subgraphs. By classifying the subgraphs formed with $T_{lookback}$ = 2, an accuracy of 0.831 was achieved. The highest weighted precision of 0.865 was achieved by classifying the subgraphs formed with $T_{lookback}$ = 1. Classifying the subgraphs formed with $T_{lookback}$ = 2 led to the highest weighted recall of 0.831 and weighted F1-score of 0.841. The highest macro- and micro-averaged values for precision (0.442 and 0.831) and F1-score (0.468 and 0.831) were all achived with subgraphs formed with $T_{lookback}$ = 2. The same applies for the micro recall (0.831), however, the highest macro recall value of 0.552 was achieved at $T_{lookback}$ = 0. Increasing the value of $T_{lookback}$ above 2 led to gradual worsening of the results across all the observed metrics, both in terms of lower averages and higher Standard Deviations.

As for the region of Portorož, Izola and Koper, Esri’s UNet model performed better than the proposed GNN-based method based on every metric. The UNet model’s performance statistics were: accuracy—0.792, weighted precision—0.827, macro precision—0.578, and micro precision—0.792. Equally, it demonstrated a consistent weighted and micro recall of 0.792, with a lower macro recall of 0.677. The F1-scores were 0.801 (weighted), 0.589 (macro), and 0.792 (micro). The proposed method achieved the best results when classification was performed of subgraphs formed with $T_{lookback}$ = 0. This resulted in an average overall accuracy and all micro measures (precision, recall, and F1-score) of 0.741. The weighted metrics were as follow: precision—0.770, recall—0.741, and F1-score—0.746. In contrast, the macro measures were quite lower, with precision at 0.520, recall at 0.577, and F1-score at 0.529. Similar to the Graz region, an overall decline in performance across all metrics was observed, beginning immediately after the $T_{lookback}$ value exceeded 0.

Table 3 and Table 4 present the CLC level 1 pixel-based classification results for the $TS_{test\text{\_}image}$, for both dataset regions, evaluated via multiple metrics. In the Graz region the GNN-based method, using $T_{lookback}$ = 1, outperformed Esri’s UNet model across all evaluation metrics. The comparative averaged scores for the GNN-based method vs. Esri’s UNet model were as follow: overall accuracy (0.888 vs. 0.857), weighted precision (0.890 vs. 0.863), micro precision, weighted recall, and micro recall (all 0.888 vs. 0.857). The results highlight a significant divergence, especially in macro precision, with a substantial difference of 0.309 (0.877 vs. 0.568), and in macro recall with an equally striking difference of 0.337 (0.907 vs. 0.570). Similarly, the F1-scores—weighted, macro, and micro (0.888, 0.889, 0.888 vs. 0.857, 0.567, 0.857) - showed a substantial contrast, proving the superior performance of the proposed GNN-based method. The performance of the proposed method outperformed Esri’s UNet model across the majority of evaluation metrics for all the tested $T_{lookback}$ values up to and including 5. However, increasing the $T_{lookback}$ value beyond 5 resulted in a degradation of performance.

For the region of Portorož, Izola and Koper, the GNN-based method using $T_{lookback}$ = 1 demonstrated superior performance across the majority of the evaluation metrics when compared to Esri’s UNet model. The performance comparison for each averaged metric is as follows: overall accuracy (0.871 vs. 0.864), weighted precision (0.876 vs. 0.869), micro precision (0.871 vs. 0.864), weighted recall (0.871 vs. 0.864), and micro recall (0.871 vs. 0.864). A significant difference of 0.096 was observed in macro recall (0.890 vs. 0.794). The F1-score comparisons were also in favour of the proposed method with weighted, macro, and micro F1-scores being 0.872 vs. 0.862, 0.862 vs. 0.821, and 0.871 vs. 0.864, respectively. The only exception was macro precision, where the UNet model scored higher (0.862) than the proposed method using subgraphs formed with $T_{lookback}$ = 0 (0.847). Increasing the value of $T_{lookback}$ beyond 1 led to a decline in the performance of the proposed method, compared to Esri’s UNet model, for the majority of the evaluation metrics.

**Figure 12:**
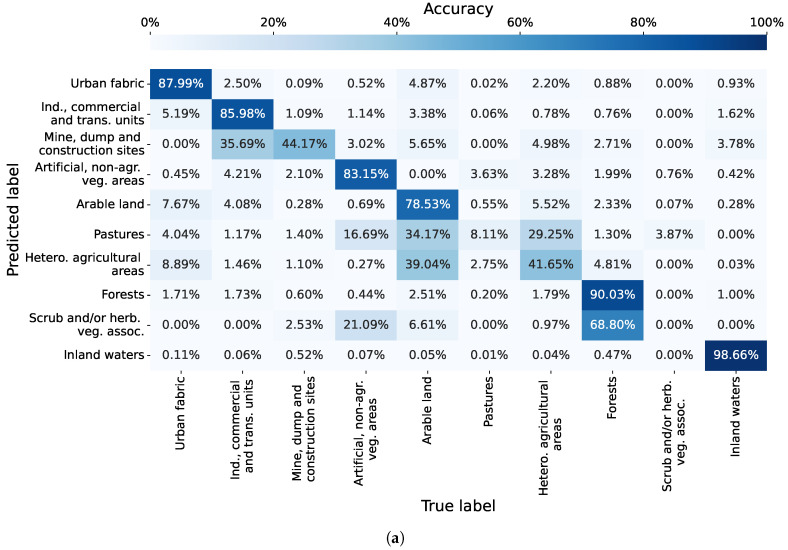

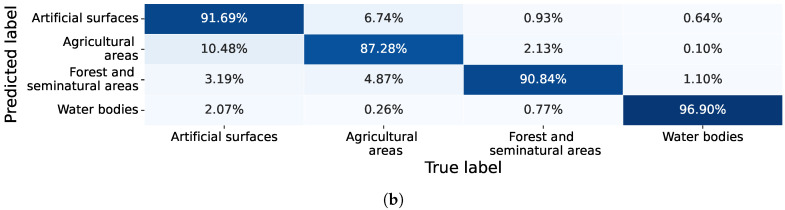
Confusion matrices for classification of the region of Graz, obtained with the best performing classification model of the proposed GNN-based method. (**a**) Confusion matrix for CLC level 2 classification. (**b**) Confusion matrix for CLC level 1 classification.

For the CLC level 2 classification of the region of Graz, the models of the proposed method, formed with $T_{lookback}$ = 2 (using spatiotemporal information from up to two previous multispectral images), secured the highest average weighted F1-score of 0.841. The model with the best F1-score of 0.846 was selected to create the confusion matrix for the classification of CLC class level 2, as depicted in Figure 12a. For the CLC level 1 classification, the highest average weighted F1-score of 0.888 was achieved by models utilising $T_{lookback}$ = 1, employing information from up to one prior multispectral image. The model boasting the top F1-score of 0.900 was used to generate the confusion matrix for CLC class level 1, showcased in Figure 12b. These confusion matrices provide insights into per-class accuracy and misclassifications exhibited by the proposed GNN-based method.

The results in the confusion matrix in Figure 12a highlight the connection between the accuracy and the number of training samples per class. High classification accuracies were achieved for classes with a large number of training samples, namely, Urban fabric (87.99% with 47,341 subgraphs), Industrial, commercial and transport units (85.98% with 24,658 subgraphs), Arable land (78.53% with 55,852 subgraphs), Forests (90.03% with 25,037 subgraphs) and Inland waters (98.66% with 4834 subgraphs). The only exception to this pattern was Artificial, non-agricultural vegetated areas with an accuracy of 83.15% and 821 training subgraphs, which is considerably less compared to other classes with high classification accuracy. The classes with a smaller number of training samples were classified poorly. Namely, Scrub and/or herbaceous vegetation associations achieved a classification accuracy of 0% by being wrongly mistaken in 68.80% of pixels for a Forest, and also to a lesser extent for other similar land cover classes. The poor accuracy results were also achieved for Mine, dump and construction sites (44.17%), Pastures (8.11%) and Heterogeneous agricultural areas (41.65%).

As for the confusion matrix for CLC level 1 classification in Figure 12b, the best performing model of the proposed GNN-based method achieved accuracy of 91.69% for Artificial surfaces, 87.28% for Agricultural areas, 90.84% for Forest and seminatural areas and 96.90% for Water bodies. The most common misclassifications occurred between Artificial surfaces and Agricultural areas.

**Figure 13:**
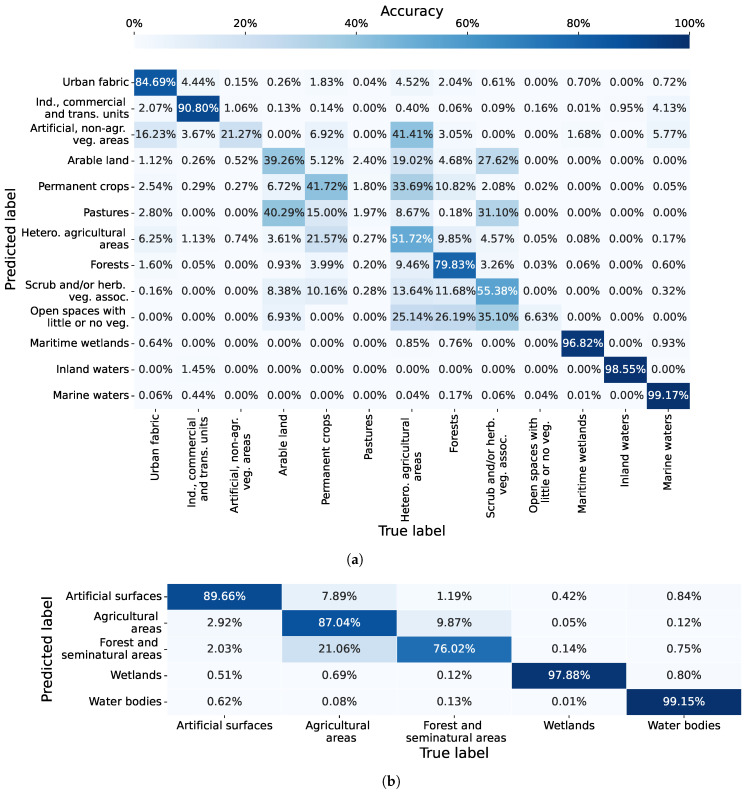
Confusion matrices for classification of the region of Portorož, Izola and Koper, obtained with the best performing classification model of the proposed GNN-based method. (**a**) Confusion matrix for CLC level 2 classification. (**b**) Confusion matrix for CLC level 1 classification.

For the CLC level 2 classification of the Portorož, Izola and Koper region, the proposed method’s models, using $T_{lookback}$ = 0 (i.e., without using any spatiotemporal information from previous multispectral images), achieved the highest average weighted F1-score of 0.764. The top-performing model, boasting an F1-score of 0.764, was utilised to construct the confusion matrix for CLC level 2 classification, as illustrated in Figure 13a. As for the CLC level 1 classification, the models with $T_{lookback}$ = 1 (which used data from one preceding multispectral image) obtained the highest average weighted F1-score of 0.872. The leading model, with an F1-score of 0.889, was employed to develop the confusion matrix for CLC level 1 classification, presented in Figure 13b.

The confusion matrix for CLC level 2 classification in Figure 13a illustrates the good performance of the proposed GNN-based method for achieving accuracy above 90% in classifying Industrial, commercial, and transport units (90.80%), Maritime wetlands (96.82%), Inland waters (98.55%), and Marine waters (99.17%). The model also performed well for Urban fabric (84.69%) and Forests (79.83%). Just as in the case of the region of Graz, the results for classes with fewer training samples (subgraphs) were suboptimal. For example, the Open spaces with little or no vegetation class, having 22 subgraphs, attained an overall classification accuracy of 6.63%. Similarly, the Pastures class, with a mere 48 training subgraphs, achieved a classification accuracy of just 1.97%.

The top-performing model of the proposed GNN-based method, as depicted in the CLC level 1 classification confusion matrix in Figure 13b, attained accuracies of 89.66% for Artificial surfaces, 87.04% for Agricultural areas, 76.02% for Forest and seminatural areas, 97.88% for Wetlands and 99.15% for Water bodies. The most common misclassifications were observed between Agricultural areas and Forest and seminatural areas, a trend observed previously in the more detailed CLC level 2 classification confusion matrix in Figure 13a.

**Figure 14:**
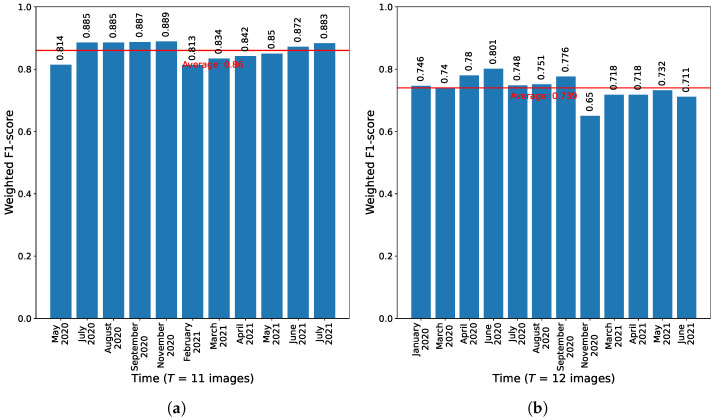
Individual weighted F1-scores for CLC level 2 classification for the consecutive images in $TS_{test\text{\_}image}$ for both regions of the dataset, obtained with the best performing corresponding classification model of the proposed GNN-based method. (**a**) Results for the 11 consecutive images of the region of Graz. (**b**) Results for the 12 consecutive images of the region of Portorož, Izola and Koper.

For both regions of the dataset, the best proposed method’s models were used to calculate individual weighted F1-scores for CLC level 2 classification of all images in the $TS_{test\text{\_}image}$, as illustrated in Figure 14. For the region of Graz these scores ranged from 0.813 to 0.889, averaging at 0.860. However, the range was wider for the region of Portorož, Izola and Koper, with scores as low as 0.65 to as high as 0.801, and an average score of 0.739. The variance in these scores for the region of Portorož, Izola and Koper was nearly double (0.0015) that of the Graz region (0.0008).

**Figure 15:**
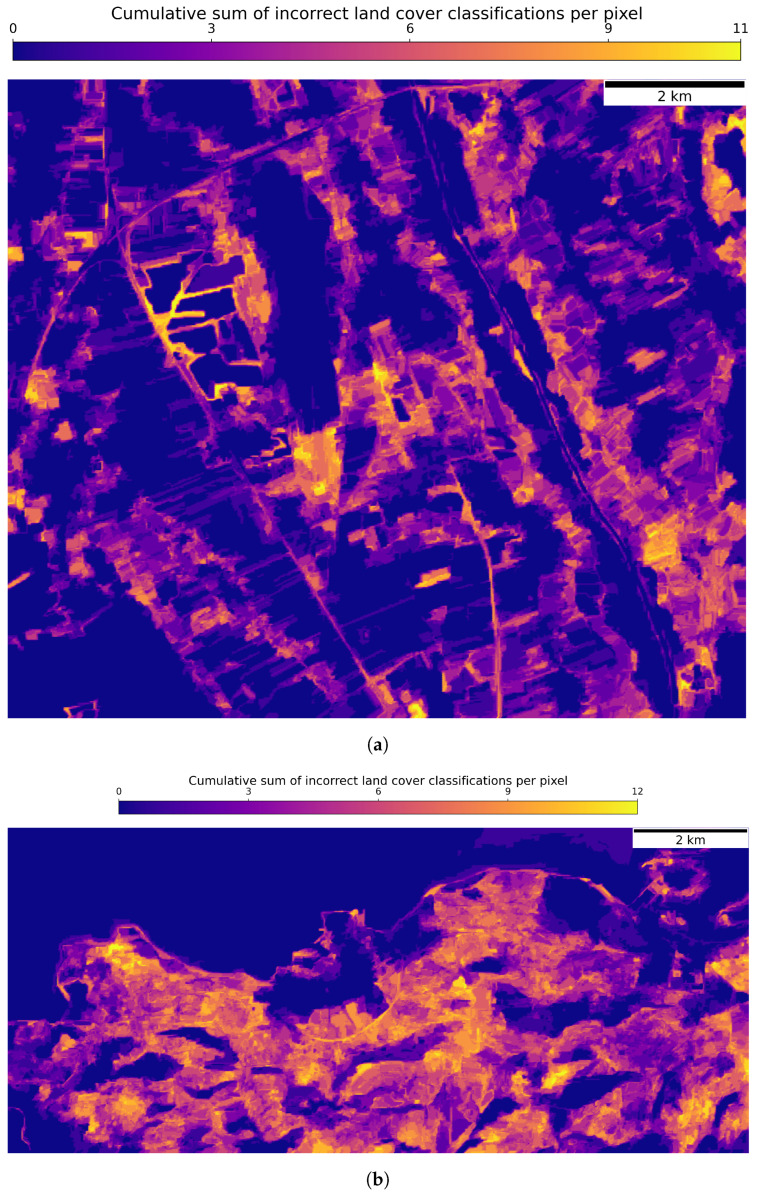
Pixel -based heatmaps of cumulative incorrect CLC level 2 classifications for both regions of the dataset, derived from classifying the $TS_{test\text{\_}image}$ with the best performing corresponding classification model of the proposed GNN-based method. The colours’ transition from dark purple to bright yellow represent the frequency of misclassifications, with intensifying brightness signifying a higher count of errors. (**a**) Heatmap for the region of Graz. (**b**) Heatmap for the region of Portorož, Izola and Koper.

**Figure 16:**
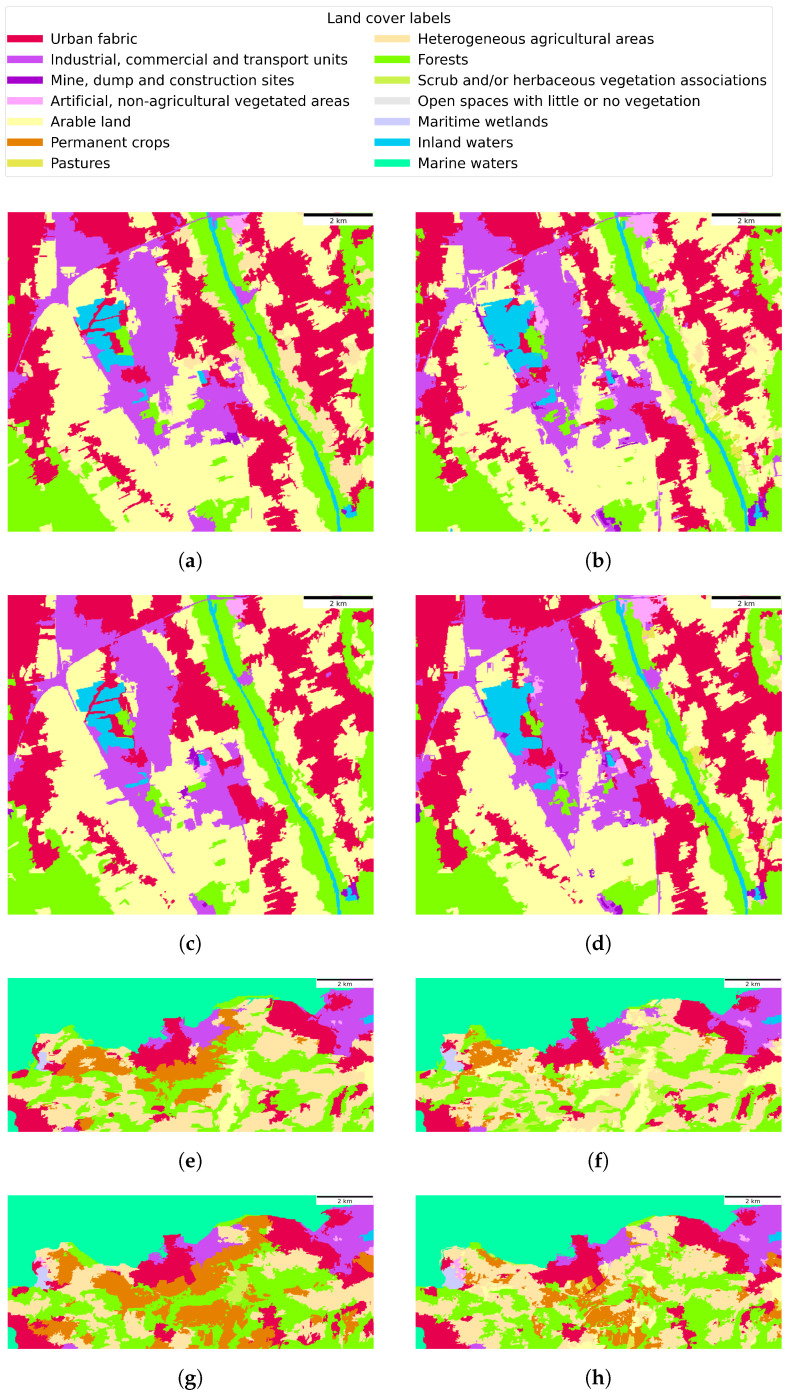
Examples of CLC level 2 ground truth ((**a**,**c**,**e**,**g**)) and predictions ((**b**,**d**,**f**,**h**)) for both regions of the dataset, obtained with the best performing corresponding classification model of the proposed GNN-based method. (**a**) Ground truth—May 2020—region of Graz, (**b**) Predicted land cover—May 2020—region of Graz, (**c**) Ground truth—June 2021—region of Graz, (**d**) Predicted land cover—June 2021—region of Graz, (**e**) Ground truth—January 2020—region of Portorož, Izola and Koper, (**f**) Predicted land cover—January 2020—region of Portorož, Izola and Koper, (**g**) Ground truth—May 2021—region of Portorož, Izola and Koper, (**h**) Predicted land cover—May 2021—region of Portorož, Izola and Koper.

The pixel-based heatmaps, depicted in Figure 15, illustrate the spatial distribution of cumulative inaccuracies in the CLC level 2 classification for both dataset regions. A subset of these land cover predictions is depicted in Figure 16. Notably, for the Graz region, the urban and industrial areas experienced the highest frequency of misclassifications, followed closely by agricultural zones. In the region of Portorož, Izola and Koper, the seminatural and agricultural landscapes were the most common sites of classification errors.

The findings demonstrate that the proposed GNN-based method outperformed Esri’s UNet model consistently across all datasets for CLC level 1 classification. However, when it comes to CLC level 2 classification, the GNN-based method’s performance was on par with that of Esri’s UNet model. While the proposed method achieved better results across all evaluation metrics in the Graz region, it underperformed in the region of Portorož, Izola and Koper.

**Figure 17:**
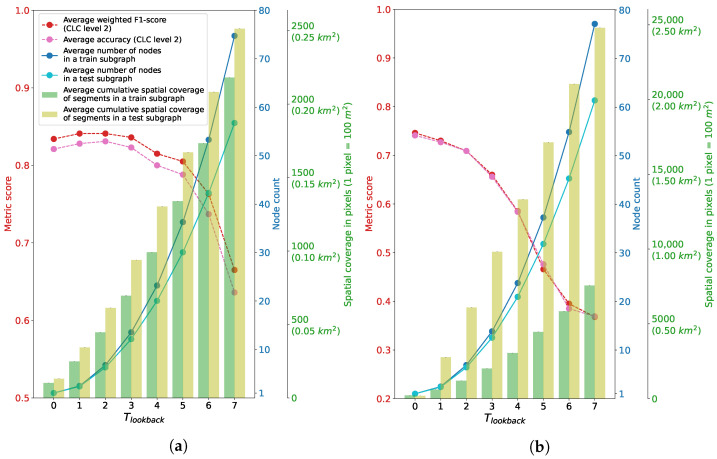
Average node count and cumulative spatial coverage (total size of all segments) in a $G_{sub}$, along with metric scores, depending on the value of $T_{lookback}$. (**a**) Subgraph-related statistics for the region of Graz. (**b**) Subgraph-related statistics for the region of Portorož, Izola and Koper.

Various factors contribute to misclassifications and performance degradation as the value of $T_{lookback}$ increases. This is evident in Figure 17, which displays statistics of subgraphs for both dataset regions. For the region of Graz, a decline in average accuracy and weighted F1-score begins when $T_{lookback}$ equals 3. This decline aligns with the average cumulative spatial coverage of segments in both training and testing subgraphs exceeding ≈0.07 km${}^{2}$. The region of Portorož, Izola and Koper displays a different pattern. The average cumulative spatial coverage of segments in the subgraphs are significantly larger than in Graz for all the tested $T_{lookback}$ values. The achieved metric scores start lower in this region in comparison to Graz. The degradation begins immediately as we increase $T_{lookback}$ above zero. In the region of Portorož, Izola and Koper, it is evident that, as the number of nodes in $G_{sub}$ increases, there is a growing disparity in the average cumulative spatial coverage of segments between the training and testing subgraphs. While this disparity could potentially cause a decline in performance, it’s worth noting that such a trend was not observed in the Graz region.

The number of nodes in a $G_{sub}$ doesn’t influence classification performance directly. However, it does control spatial coverage, which affects classification performance significantly. Hence, the primary cause of poor classification performance are overly large spatial regions captured by larger subgraphs through time. Such expansive regions can contain conflicting segment information, resulting in misclassifications. This issue is mainly a result of segmentation parameters, although an increase in the value of $T_{lookback}$ also plays a contributing role. The described issue with conflicting segment information is particularly evident for classes like Industrial, commercial and transport units, Mine, dump and construction sites, as well as Arable land, Pastures, and Heterogeneous agricultural areas, as visualised by the confusion matrices in Figure 12a and Figure 13a.

**Figure 18:**
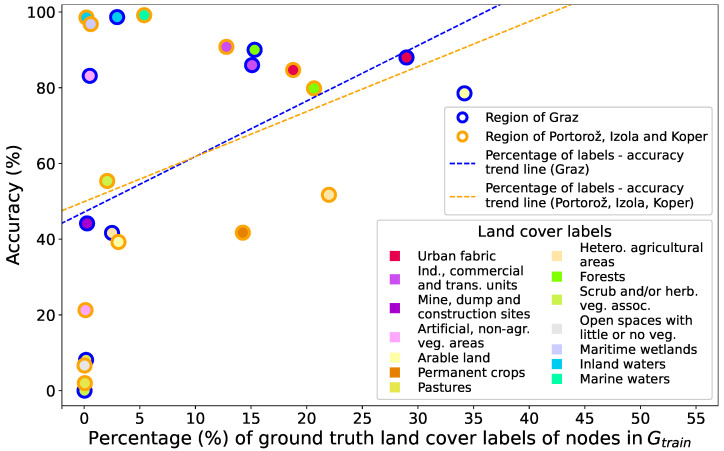
Class-specific CLC level 2 classification accuracies (sourced from the confusion matrices in Figure 12a and Figure 13a) for both dataset regions in relation to the distribution of ground truth land cover labels in $G_{train}$, derived from Figure 10a,c.

Dataset imbalance impacts the class-specific accuracies for both dataset regions, as demonstrated in Figure 18. The trend lines suggest higher accuracies are associated with classes that occupy a larger proportion of node labels within the $G_{train}$. However, several classes have emerged as exceptions to this rule. For instance, in the Graz region, only 2.96% of nodes in the $G_{train}$ are labelled as Inland waters, but this still results in an accuracy of 98.66%. To achieve better performance, complex classes such as Pastures and Open spaces with little or no vegetation require a greater quantity of nodes with corresponding labels.

The proposed method faces certain limitations. Firstly, the segmentation parameters should be selected carefully, because small segments result in *G* containing a large set of nodes. This leads to the increase of the processing time required to classify all target nodes within the *G*. Secondly, a smaller value of the τ parameter during graph construction leads to more edges being established between nodes, consequently enlarging the $G_{sub}$ of each individual $v_{target}$. Larger $G_{sub}$ can potentially capture a too broad spatial region through time, and may introduce conflicting information from the segments, thereby undermining the classification results. Additionally, larger subgraphs result in slower training and extended inference times, which limits the efficiency of the proposed GNN-based method.

## 6. Conclusions

This paper presented a novel spatiotemporal method for an object-based land cover classification of satellite imagery using a GNN. A 4-year intermonthly land cover ground truth dataset of Sentinel-2 imagery was created for two regions: Graz in Austria, and the region of Portorož, Izola and Koper in Slovenia. This newly created dataset, classified in accordance with the CLC level 2 nomenclature, was utilised to evaluate the performance of the proposed method. For testing purposes, the method’s classification pipeline used EfficientNetV2-S for image feature extraction, and the GraphSAGE algorithm with LSTM aggregation for target node classification.

For the CLC level 2 classification of the region of Graz, the proposed method outperformed the state-of-the-art UNet model across all evaluation metrics. The proposed method yielded the best average weighted F1-score of 0.841 and an accuracy of 0.831, which surpassed the UNet model’s results of 0.824 and 0.818, respectively. For the classification of the region of Portorož, Izola and Koper, the UNet model achieved better results compared to the proposed method. The proposed method achieved a peak average weighted F1-score of 0.746 and accuracy of 0.741, underperforming the UNet model’s results of 0.801 and 0.792, respectively.

However, by combining the CLC level 2 into the predefined CLC level 1 classification, the proposed method demonstrated far superior performance for both regions. The proposed method yielded the best average weighted F1-score and accuracy of 0.888 for the Graz region, outperforming the UNet model’s performance, which achieved a score of 0.857 for both metrics. Similarly, for the region of Portorož, Izola and Koper, the proposed method achieved the highest average weighted F1-score of 0.872 and accuracy of 0.871, beating the UNet model, which scored 0.862 and 0.864, respectively.

The findings indicate that the classification performance of the proposed GNN-method starts to decline when an excessive amount of spatiotemporal information from numerous preceding multispectral images is included in the subgraphs. The proposed method exhibits limitations related to the choice of segmentation and graph construction parameters.

In the future, the proposed method’s parameter of maximum distance of nodes based on input edges towards the target node will become adaptable using the multispectral data. The proposed method will also be extended with additional types of input data, and modified to perform object-based regression tasks, e.g., particulate matter trends.

## Figures and Tables

**Table 1:** Accuracy, precision, recall and F1-score for CLC level 2 classification of $TS_{test\text{\_}image}$ for a region of Graz. The best result for each metric is marked in bold.

		Metric									
Classification	$T_{\mathrm{lookback}}$	Accuracy	Precision			Recall			F1-Score		
Method			Weighted	Macro	Micro	Weighted	Macro	Micro	Weighted	Macro	Micro
Esri’s UNet	/	0.818	0.841	0.349	0.818	0.818	0.465	0.818	0.824	0.371	0.818
Proposed GNN-based method	0	0.821 ± 0.016	0.862 ± 0.004	0.432 ± 0.021	0.821 ± 0.016	0.821 ± 0.016	**0.552 ± 0.024**	0.821 ± 0.016	0.834 ± 0.012	0.458 ± 0.020	0.821 ± 0.016
	1	0.828 ± 0.013	**0.865 ± 0.005**	0.441 ± 0.030	0.828 ± 0.013	0.828 ± 0.013	0.535 ± 0.029	0.828 ± 0.013	0.841 ± 0.010	0.465 ± 0.032	0.828 ± 0.013
	2	**0.831 ± 0.004**	0.858 ± 0.003	**0.442 ± 0.031**	**0.831 ± 0.004**	**0.831 ± 0.004**	0.534 ± 0.040	**0.831 ± 0.004**	**0.841 ± 0.003**	**0.468 ± 0.034**	**0.831 ± 0.004**
	3	0.823 ± 0.016	0.855 ± 0.005	0.409 ± 0.009	0.823 ± 0.016	0.823 ± 0.016	0.509 ± 0.017	0.823 ± 0.016	0.836 ± 0.010	0.432 ± 0.012	0.823 ± 0.016
	4	0.800 ± 0.015	0.840 ± 0.015	0.392 ± 0.009	0.800 ± 0.015	0.800 ± 0.015	0.494 ± 0.016	0.800 ± 0.015	0.815 ± 0.014	0.412 ± 0.012	0.800 ± 0.015
	5	0.788 ± 0.020	0.836 ± 0.010	0.401 ± 0.027	0.788 ± 0.020	0.788 ± 0.020	0.508 ± 0.028	0.788 ± 0.020	0.805 ± 0.016	0.422 ± 0.030	0.788 ± 0.020
	6	0.737 ± 0.030	0.810 ± 0.022	0.386 ± 0.033	0.737 ± 0.030	0.737 ± 0.030	0.482 ± 0.058	0.737 ± 0.030	0.763 ± 0.030	0.393 ± 0.040	0.737 ± 0.030
	7	0.636 ± 0.085	0.737 ± 0.075	0.391 ± 0.045	0.636 ± 0.085	0.636 ± 0.085	0.478 ± 0.044	0.636 ± 0.085	0.665 ± 0.091	0.395 ± 0.050	0.636 ± 0.085

**Table 2:** Accuracy, precision, recall and F1-score for CLC level 2 classification of $TS_{test\text{\_}image}$ for a region of Portorož, Izola and Koper. The best result for each metric is marked in bold.

		Metric									
Classification	$T_{\mathrm{lookback}}$	Accuracy	Precision			Recall			F1-Score		
Method			Weighted	Macro	Micro	Weighted	Macro	Micro	Weighted	Macro	Micro
Esri’s UNet	/	**0.792**	**0.827**	**0.578**	**0.792**	**0.792**	**0.677**	**0.792**	**0.801**	**0.589**	**0.792**
Proposed GNN-based method	0	0.741 ± 0.021	0.770 ± 0.011	0.520 ± 0.020	0.741 ± 0.021	0.741 ± 0.021	0.577 ± 0.014	0.741 ± 0.021	0.746 ± 0.019	0.529 ± 0.021	0.741 ± 0.021
	1	0.727 ± 0.022	0.762 ± 0.013	0.512 ± 0.018	0.727 ± 0.022	0.727 ± 0.022	0.575 ± 0.013	0.727 ± 0.022	0.730 ± 0.022	0.522 ± 0.015	0.727 ± 0.022
	2	0.709 ± 0.012	0.736 ± 0.009	0.491 ± 0.029	0.709 ± 0.012	0.709 ± 0.012	0.561 ± 0.011	0.709 ± 0.012	0.709 ± 0.011	0.504 ± 0.021	0.709 ± 0.012
	3	0.656 ± 0.051	0.714 ± 0.021	0.439 ± 0.038	0.656 ± 0.051	0.656 ± 0.051	0.531 ± 0.029	0.656 ± 0.051	0.660 ± 0.047	0.448 ± 0.051	0.656 ± 0.051
	4	0.584 ± 0.037	0.674 ± 0.022	0.369 ± 0.023	0.584 ± 0.037	0.584 ± 0.037	0.492 ± 0.020	0.584 ± 0.037	0.585 ± 0.033	0.362 ± 0.027	0.584 ± 0.037
	5	0.476 ± 0.042	0.595 ± 0.043	0.311 ± 0.028	0.476 ± 0.042	0.476 ± 0.042	0.421 ± 0.023	0.476 ± 0.042	0.466 ± 0.049	0.285 ± 0.025	0.476 ± 0.042
	6	0.385 ± 0.089	0.557 ± 0.044	0.249 ± 0.029	0.385 ± 0.089	0.385 ± 0.089	0.279 ± 0.053	0.385 ± 0.089	0.395 ± 0.090	0.199 ± 0.036	0.385 ± 0.089
	7	0.369 ± 0.046	0.463 ± 0.040	0.192 ± 0.018	0.369 ± 0.046	0.369 ± 0.046	0.206 ± 0.021	0.369 ± 0.046	0.368 ± 0.035	0.157 ± 0.020	0.369 ± 0.046

**Table 3:** Accuracy, precision, recall and F1-score for CLC level 1 classification of $TS_{test\text{\_}image}$ for a region of Graz. The best result for each metric is marked in bold.

		Metric									
Classification	$T_{\mathrm{lookback}}$	Accuracy	Precision			Recall			F1-Score		
Method			Weighted	Macro	Micro	Weighted	Macro	Micro	Weighted	Macro	Micro
Esri’s UNet	/	0.857	0.863	0.568	0.857	0.857	0.570	0.857	0.857	0.567	0.857
Proposed GNN-based method	0	0.881 ± 0.007	0.883 ± 0.006	0.867 ± 0.006	0.881 ± 0.007	0.881 ± 0.007	0.901 ± 0.010	0.881 ± 0.007	0.880 ± 0.008	0.881 ± 0.007	0.881 ± 0.007
	1	**0.888 ± 0.006**	**0.890 ± 0.006**	**0.877 ± 0.008**	**0.888 ± 0.006**	**0.888 ± 0.006**	**0.907 ± 0.008**	**0.888 ± 0.006**	**0.888 ± 0.007**	**0.889 ± 0.006**	**0.888 ± 0.006**
	2	0.886 ± 0.003	0.888 ± 0.002	0.872 ± 0.009	0.886 ± 0.003	0.886 ± 0.003	0.906 ± 0.007	0.886 ± 0.003	0.886 ± 0.003	0.886 ± 0.002	0.886 ± 0.003
	3	0.882 ± 0.009	0.883 ± 0.008	0.860 ± 0.010	0.882 ± 0.009	0.882 ± 0.009	0.903 ± 0.009	0.882 ± 0.009	0.882 ± 0.009	0.878 ± 0.009	0.882 ± 0.009
	4	0.866 ± 0.015	0.868 ± 0.014	0.846 ± 0.017	0.866 ± 0.015	0.866 ± 0.015	0.885 ± 0.026	0.866 ± 0.015	0.865 ± 0.015	0.861 ± 0.020	0.866 ± 0.015
	5	0.859 ± 0.011	0.863 ± 0.010	0.838 ± 0.012	0.859 ± 0.011	0.859 ± 0.011	0.884 ± 0.011	0.859 ± 0.011	0.859 ± 0.011	0.855 ± 0.013	0.859 ± 0.011
	6	0.822 ± 0.027	0.832 ± 0.024	0.789 ± 0.035	0.822 ± 0.027	0.822 ± 0.027	0.839 ± 0.031	0.822 ± 0.027	0.821 ± 0.028	0.798 ± 0.042	0.822 ± 0.027
	7	0.756 ± 0.071	0.770 ± 0.065	0.723 ± 0.069	0.756 ± 0.071	0.756 ± 0.071	0.796 ± 0.059	0.756 ± 0.071	0.756 ± 0.074	0.738 ± 0.074	0.756 ± 0.071

**Table 4:** Accuracy, precision, recall and F1-score for CLC level 1 classification of $TS_{test\text{\_}image}$ for a region of Portorož, Izola and Koper. The best result for each metric is marked in bold.

		Metric									
Classification	$T_{\mathrm{lookback}}$	Accuracy	Precision			Recall			F1-Score		
Method			Weighted	Macro	Micro	Weighted	Macro	Micro	Weighted	Macro	Micro
Esri’s UNet	/	0.864	0.869	**0.862**	0.864	0.864	0.794	0.864	0.862	0.821	0.864
Proposed GNN-based method	0	0.864 ± 0.023	0.873 ± 0.011	0.847 ± 0.014	0.864 ± 0.023	0.864 ± 0.023	0.882 ± 0.016	0.864 ± 0.023	0.864 ± 0.024	0.859 ± 0.018	0.864 ± 0.023
	1	**0.871 ± 0.014**	**0.876 ± 0.010**	0.844 ± 0.020	**0.871 ± 0.014**	**0.871 ± 0.014**	**0.890 ± 0.010**	**0.871 ± 0.014**	**0.872 ± 0.013**	**0.862 ± 0.016**	**0.871 ± 0.014**
	2	0.861 ± 0.012	0.865 ± 0.009	0.830 ± 0.019	0.861 ± 0.012	0.861 ± 0.012	0.882 ± 0.007	0.861 ± 0.012	0.861 ± 0.012	0.850 ± 0.016	0.861 ± 0.012
	3	0.841 ± 0.026	0.843 ± 0.024	0.793 ± 0.045	0.841 ± 0.026	0.841 ± 0.026	0.859 ± 0.022	0.841 ± 0.026	0.840 ± 0.026	0.814 ± 0.047	0.841 ± 0.026
	4	0.793 ± 0.027	0.803 ± 0.022	0.712 ± 0.037	0.793 ± 0.027	0.793 ± 0.027	0.814 ± 0.019	0.793 ± 0.027	0.788 ± 0.030	0.722 ± 0.040	0.793 ± 0.027
	5	0.734 ± 0.033	0.750 ± 0.026	0.646 ± 0.039	0.734 ± 0.033	0.734 ± 0.033	0.752 ± 0.028	0.734 ± 0.033	0.730 ± 0.025	0.644 ± 0.046	0.734 ± 0.033
	6	0.623 ± 0.072	0.710 ± 0.033	0.571 ± 0.034	0.623 ± 0.072	0.623 ± 0.072	0.586 ± 0.088	0.623 ± 0.072	0.638 ± 0.056	0.515 ± 0.042	0.623 ± 0.072
	7	0.572 ± 0.067	0.615 ± 0.050	0.484 ± 0.047	0.572 ± 0.067	0.572 ± 0.067	0.461 ± 0.048	0.572 ± 0.067	0.572 ± 0.063	0.451 ± 0.053	0.572 ± 0.067

## Data Availability

The data presented in this paper are available on request from the corresponding author. The data are not publicly available due to ongoing analysis and publication commitments.

## References

[1] Bhandari A., Joshi R., Thapa M.S., Sharma R.P., Rauniyar S.K. (2022). Land Cover Change and Its Impact in Crop Yield: A Case Study from Western Nepal. Sci. World J..

[2] Hussain S., Mubeen M., Ahmad A., Majeed H., Qaisrani S., Hammad H., Amjad M., Ahmad I., Fahad S., Ahmad N. (2022). Assessment of land use/land cover changes and its effect on land surface temperature using remote sensing techniques in Southern Punjab, Pakistan. Environ. Sci. Pollut. Res..

[3] Som-ard J., Immitzer M., Vuolo F., Ninsawat S., Atzberger C. (2022). Mapping of crop types in 1989, 1999, 2009 and 2019 to assess major land cover trends of the Udon Thani Province, Thailand. Comput. Electron. Agric..

[4] Koetz B., Morsdorf F., van der Linden S., Curt T., Allgöwer B. (2008). Multi-source land cover classification for forest fire management based on imaging spectrometry and LiDAR data. For. Ecol. Manag..

[5] Hao L., van Westen C., Rajaneesh A., Sajinkumar K., Martha T.R., Jaiswal P. (2022). Evaluating the relation between land use changes and the 2018 landslide disaster in Kerala, India. CATENA.

[6] Shuaishuai J., Yang C., Wang M., Failler P. (2022). Heterogeneous Impact of Land-Use on Climate Change: Study From a Spatial Perspective. Front. Environ. Sci..

[7] Aslam S., Chak Y.C., Hussain Jaffery M., Varatharajoo R., Ahmad Ansari E. (2023). Model predictive control for Takagi–Sugeno fuzzy model-based Spacecraft combined energy and attitude control system. Adv. Space Res..

[8] Yahya N., Varatharajoo R., Mohd Harithuddin A.S. (2020). Satellite Formation Flying Relative Geodesic and Latitudinal Error Measures. J. Aeronaut. Astronaut. Aviat. Ser. A.

[9] Li J., Chen B. (2020). Global Revisit Interval Analysis of Landsat-8 -9 and Sentinel-2A -2B Data for Terrestrial Monitoring. Sensors.

[10] Yin J., Dong J., Hamm N.A., Li Z., Wang J., Xing H., Fu P. (2021). Integrating remote sensing and geospatial big data for urban land use mapping: A review. Int. J. Appl. Earth Obs. Geoinf..

[11] Hansen M.C., Loveland T.R. (2012). A review of large area monitoring of land cover change using Landsat data. Remote Sens. Environ..

[12] Phiri D., Simwanda M., Salekin S., Nyirenda V.R., Murayama Y., Ranagalage M. (2020). Sentinel-2 Data for Land Cover/Use Mapping: A Review. Remote Sens..

[13] Zhu Z., Wang S., Woodcock C.E. (2015). Improvement and expansion of the Fmask algorithm: Cloud, cloud shadow, and snow detection for Landsats 4–7, 8, and Sentinel 2 images. Remote Sens. Environ..

[14] Frantz D., Haß E., Uhl A., Stoffels J., Hill J. (2018). Improvement of the Fmask algorithm for Sentinel-2 images: Separating clouds from bright surfaces based on parallax effects. Remote Sens. Environ..

[15] Dupuy S., Gaetano R. (2020). Reunion Island-2019, Land Cover Map (Spot6/7)-1.5 m. https://dataverse.cirad.fr/dataset.xhtml?persistentId=doi:10.18167/DVN1/YZJQ7Q.

[16] Censi A.M., Ienco D., Gbodjo Y.J.E., Pensa R.G., Interdonato R., Gaetano R. (2021). Attentive Spatial Temporal Graph CNN for Land Cover Mapping From Multi Temporal Remote Sensing Data. IEEE Access.

[17] Heymann Y., Steenmans C., Croisille G., Bossard M., Lenco M., Wyatt B., Jean-Louis W., O’Brian C., Cornaert M.-H., Nicolas S. (1994). Corine Land Cover Technical Guide, Part I.

[18] (2018). Copernicus Land Monitoring Service 2018. https://land.copernicus.eu/pan-european/corine-land-cover/clc2018.

[19] Soukup T., Feranec J., Hazeu G., Jaffrain G., Jindrova M., Kopecky M., Orlitova E., Feranec J., Soukup T., Hazeu G., Jaffrain G. (2016). Chapter 10 CORINE Land Cover 1990 (CLC1990): Analysis and Assessment: CORINE Land Cover Data. European Landscape Dynamics.

[20] Buttner G., Feranec J., Jaffrain G., Mari L., Maucha G., Soukup T. (2004). The CORINE land cover 2000 project. EARSeL Eproceedings.

[21] Soukup T., Feranec J., Hazeu G., Jaffrain G., Jindrova M., Kopecky M., Orlitova E., Feranec J., Soukup T., Hazeu G., Jaffrain G. (2016). Chapter 12 CORINE Land Cover 2006 (CLC2006): Analysis and Assessment: CORINE Land Cover Data. European Landscape Dynamics.

[22] Soukup T., Büttner G., Feranec J., Hazeu G., Jaffrain G., Jindrova M., Kopecky M., Orlitova E., Feranec J., Soukup T., Hazeu G., Jaffrain G. (2016). Chapter 13 CORINE Land Cover 2012 (CLC2012): Analysis and Assessment: CORINE Land Cover Data. European Landscape Dynamics.

[23] Aune-Lundberg L., Strand G.H. (2020). The content and accuracy of the CORINE Land Cover dataset for Norway. Int. J. Appl. Earth Obs. Geoinf..

[24] Eurostat (2021). LUCAS—EU Land Use and Cover Area Survey—2021 Edition.

[25] Landa M., Brodský L., Halounová L., Bouček T., Pešek O. (2022). Open Geospatial System for LUCAS In Situ Data Harmonization and Distribution. ISPRS Int. J. Geo-Inf..

[26] Brown C., Brumby S., Guzder-Williams B., Birch T., Hyde S., Mazzariello J., Czerwinski W., Pasquarella V., Haertel R., Ilyushchenko S. (2022). Dynamic World, Near real-time global 10 m land use land cover mapping. Sci. Data.

[27] Hofierka J., Gallay M., Onačillová K., Hofierka J. (2020). Physically-based land surface temperature modeling in urban areas using a 3-D city model and multispectral satellite data. Urban Clim..

[28] Li S., Xiong L., Tang G., Strobl J. (2020). Deep learning-based approach for landform classification from integrated data sources of digital elevation model and imagery. Geomorphology.

[29] Gaur S., Singh R. (2023). A Comprehensive Review on Land Use/Land Cover (LULC) Change Modeling for Urban Development: Current Status and Future Prospects. Sustainability.

[30] Gašparović M., Jogun T. (2018). The Effect of Fusing Sentinel-2 Bands on Land-Cover Classification. Int. J. Remote Sens..

[31] Gómez C., White J.C., Wulder M.A. (2016). Optical remotely sensed time series data for land cover classification: A review. ISPRS J. Photogramm. Remote Sens..

[32] Zhang C., Yue P., Tapete D., Shangguan B., Wang M., Wu Z. (2020). A multi-level context-guided classification method with object-based convolutional neural network for land cover classification using very high resolution remote sensing images. Int. J. Appl. Earth Obs. Geoinf..

[33] Mongus D., Žalik B. (2018). Segmentation schema for enhancing land cover identification: A case study using Sentinel 2 data. Int. J. Appl. Earth Obs. Geoinf..

[34] Yang C., Rottensteiner F., Heipke C. (2021). A hierarchical deep learning framework for the consistent classification of land use objects in geospatial databases. ISPRS J. Photogramm. Remote Sens..

[35] Fitton D., Laurens E., Hongkarnjanakul N., Schwob C., Mezeix L. (2022). Land cover classification through Convolutional Neural Network model assembly: A case study of a local rural area in Thailand. Remote Sens. Appl. Soc. Environ..

[36] Fu J., Yi X., Wang G., Mo L., Wu P., Kapula K.E. (2022). Research on Ground Object Classification Method of High Resolution Remote-Sensing Images Based on Improved DeeplabV3+. Sensors.

[37] Li M., Lu Y., Cao S., Wang X., Xie S. (2023). A Hyperspectral Image Classification Method Based on the Nonlocal Attention Mechanism of a Multiscale Convolutional Neural Network. Sensors.

[38] Li J., Wang H., Zhang A., Liu Y. (2022). Semantic Segmentation of Hyperspectral Remote Sensing Images Based on PSE-UNet Model. Sensors.

[39] Abidi A., Ienco D., Abbes A.B., Farah I.R. (2023). Combining 2D encoding and convolutional neural network to enhance land cover mapping from Satellite Image Time Series. Eng. Appl. Artif. Intell..

[40] Qiu C., Mou L., Schmitt M., Zhu X.X. (2019). Local climate zone-based urban land cover classification from multi-seasonal Sentinel-2 images with a recurrent residual network. ISPRS J. Photogramm. Remote Sens..

[41] Dantas C.F., Marcos D., Ienco D. (2023). Counterfactual Explanations for Land Cover Mapping in a Multi-class Setting. arXiv.

[42] Chen B., Zheng H., Wang L., Hellwich O., Chen C., Yang L., Liu T., Luo G., Bao A., Chen X. (2022). A joint learning Im-BiLSTM model for incomplete time-series Sentinel-2A data imputation and crop classification. Int. J. Appl. Earth Obs. Geoinf..

[43] Jiang Z., Yang S., Liu Z., Xu Y., Xiong Y., Qi S., Pang Q., Xu J., Liu F., Xu T. (2022). Coupling machine learning and weather forecast to predict farmland flood disaster: A case study in Yangtze River basin. Environ. Model. Softw..

[44] Aamir M., Ali T., Irfan M., Shaf A., Azam M.Z., Glowacz A., Brumercik F., Glowacz W., Alqhtani S., Rahman S. (2021). Natural Disasters Intensity Analysis and Classification Based on Multispectral Images Using Multi-Layered Deep Convolutional Neural Network. Sensors.

[45] Siddiqui M.K., Imran M., Ahmad A. (2016). On Zagreb indices, Zagreb polynomials of some nanostar dendrimers. Appl. Math. Comput..

[46] Ahmad A., Bača M., Siddiqui M.K. (2013). On Edge Irregular Total Labeling of Categorical Product of Two Cycles. Theory Comput. Syst..

[47] Azeem M., Imran M., Nadeem M.F. (2022). Sharp bounds on partition dimension of hexagonal Möbius ladder. J. King Saud Univ. Sci..

[48] Pang H.E., Biljecki F. (2022). 3D building reconstruction from single street view images using deep learning. Int. J. Appl. Earth Obs. Geoinf..

[49] Ding Y., Zhang Z., Zhao X., Hong D., Li W., Cai W., Zhan Y. (2022). AF2GNN: Graph convolution with adaptive filters and aggregator fusion for hyperspectral image classification. Inf. Sci..

[50] Dosovitskiy A., Beyer L., Kolesnikov A., Weissenborn D., Zhai X., Unterthiner T., Dehghani M., Minderer M., Heigold G., Gelly S. An Image is Worth 16x16 Words: Transformers for Image Recognition at Scale. Proceedings of the International Conference on Learning Representations.

[51] Tan M., Le Q.V. EfficientNetV2: Smaller Models and Faster Training. Proceedings of the 38th International Conference on Machine Learning.

[52] Tan M., Le Q.V. (2020). EfficientNet: Rethinking Model Scaling for Convolutional Neural Networks. arXiv.

[53] He K., Zhang X., Ren S., Sun J. Deep Residual Learning for Image Recognition. Proceedings of the IEEE Conference on Computer Vision and Pattern Recognition (CVPR).

[54] Xie S., Girshick R., Dollar P., Tu Z., He K. (2017). Aggregated Residual Transformations for Deep Neural Networks. arXiv.

[55] Szegedy C., Vanhoucke V., Ioffe S., Shlens J., Wojna Z. (2016). Rethinking the Inception Architecture for Computer Vision. empharXiv.

[56] Huang G., Liu Z., van der Maaten L., Weinberger K. (2017). Densely Connected Convolutional Networks. arXiv.

[57] Veličković P., Cucurull G., Casanova A., Romero A., Liò P., Bengio Y. (2017). Graph Attention Networks. arXiv.

[58] Hamilton W., Ying Z., Leskovec J. Inductive Representation Learning on Large Graphs. Proceedings of the 31st Conference on Neural Information Processing Systems (NeurIPS 2017).

[59] Kaiser P., Wegner J., Lucchi A., Jaggi M., Hofmann T., Schindler K. (2017). Learning Aerial Image Segmentation From Online Maps. IEEE Trans. Geosci. Remote Sens..

[60] Nasir S.M., Kamran K.V., Blaschke T., Karimzadeh S. (2022). Change of land use / land cover in kurdistan region of Iraq: A semi-automated object-based approach. Remote Sens. Appl. Soc. Environ..

[61] Liu T., Abd-Elrahman A. (2018). Deep convolutional neural network training enrichment using multi-view object-based analysis of Unmanned Aerial systems imagery for wetlands classification. ISPRS J. Photogramm. Remote Sens..

[62] Herawan A., Julzarika A., Hakim P., Asti E. (2021). Object-Based on Land Cover Classification on LAPAN-A3 Satellite Imagery Using Tree Algorithm (Case Study: Rote Island). Int. J. Adv. Sci. Eng. Inf. Technol.y.

[63] Vizzari M. (2022). PlanetScope, Sentinel-2, and Sentinel-1 Data Integration for Object-Based Land Cover Classification in Google Earth Engine. Remote Sens..

[64] Hedayati A., Vahidnia M.H., Behzadi S. (2022). Paddy lands detection using Landsat-8 satellite images and object-based classification in Rasht city, Iran. Egypt. J. Remote Sens. Space Sci..

[65] Kipf T.N., Welling M. Semi-Supervised Classification with Graph Convolutional Networks. Proceedings of the 5th International Conference on Learning Representations, ICLR 2017.

[66] Zhao L., Song Y., Zhang C., Liu Y., Wang P., Lin T., Deng M., Li H. (2020). T-GCN: A Temporal Graph Convolutional Network for Traffic Prediction. IEEE Trans. Intell. Transp. Syst..

[67] Zhang Z., Huang J., Tan Q. (2020). SR-HGAT: Symmetric Relations Based Heterogeneous Graph Attention Network. IEEE Access.

[68] Hu B., Guo K., Wang X., Zhang J., Zhou D. (2022). RRL-GAT: Graph Attention Network-Driven Multilabel Image Robust Representation Learning. IEEE Internet Things J..

[69] Ying R., He R., Chen K., Eksombatchai P., Hamilton W.L., Leskovec J. Graph Convolutional Neural Networks for Web-Scale Recommender Systems. Proceedings of the 24th ACM SIGKDD International Conference on Knowledge Discovery & Data Mining, KDD 2018.

[70] Zhao W., Peng S., Chen J., Peng R. (2022). Contextual-Aware Land Cover Classification With U-Shaped Object Graph Neural Network. IEEE Geosci. Remote Sens. Lett..

[71] Achanta R., Shaji A., Smith K., Lucchi A., Fua P., Süsstrunk S. (2012). SLIC Superpixels Compared to State-of-the-Art Superpixel Methods. IEEE Trans. Pattern Anal. Mach. Intell..

[72] Stutz D., Hermans A., Leibe B. (2018). Superpixels: An evaluation of the state-of-the-art. Comput. Vis. Image Underst..

[73] Felzenszwalb P.F., Huttenlocher D.P. (2004). Efficient Graph-Based Image Segmentation. Int. J. Comput. Vis..

[74] Kingma D., Ba J. Adam: A Method for Stochastic Optimization. Proceedings of the International Conference on Learning Representations (ICLR 2015).

[75] Grandini M., Bagli E., Visani G. (2020). Metrics for Multi-Class Classification: An Overview. arXiv.

[76] Lever J., Krzywinski M., Altman N. (2016). Points of Significance: Classification evaluation. Nat. Methods.

